# Targeting of the G9a, DNMT1 and UHRF1 epigenetic complex as an effective strategy against pancreatic ductal adenocarcinoma

**DOI:** 10.1186/s13046-024-03268-5

**Published:** 2025-01-15

**Authors:** Daniel Oyon, Amaya Lopez-Pascual, Borja Castello-Uribe, Iker Uriarte, Giulia Orsi, Sofia Llorente, Jasmin Elurbide, Elena Adan-Villaescusa, Emiliana Valbuena-Goiricelaya, Ainara Irigaray-Miramon, Maria Ujue Latasa, Luz A. Martinez-Perez, Luca Reggiani Bonetti, Felipe Prosper, Mariano Ponz-Sarvise, Silvestre Vicent, Antonio Pineda-Lucena, David Ruiz-Clavijo, Bruno Sangro, Urko Aguirre Larracoechea, Tian V. Tian, Andrea Casadei-Gardini, Irene Amat, Maria Arechederra, Carmen Berasain, Jesus M. Urman, Matias A. Avila, Maite G. Fernandez-Barrena

**Affiliations:** 1 Department of Gastroenterology, Galdakao-Usansolo Hospital, Galdakao, Spain; 2 Biobizkaia Health Research Institute, Bizkaia, Spain; 3 Hepatology Laboratory, Solid Tumors Program, CIMA, CCUN, University of Navarra, Pamplona, Spain; 4 Navarra Institute for Health Research, IdiSNA, Pamplona, Spain; 5 CIBERehd, Instituto de Salud Carlos III, Madrid, Spain; 6 Oncology Department, University Hospital of Modena, Modena, Italy; 7 Department of Oncology, IRCCS San Raffaele Scientific Institute Hospital, Vita-Salute San Raffaele University, Milan, Italy; 8 Preclinical and Translational Research Program, Vall d’Hebron Institute of Oncology (VHIO), Barcelona, Spain; 9 Universidad de Guadalajara Centro Universitario de Ciencias de La Salud, Guadalajara, Mexico; 10 Department of Medical and Surgical Sciences for Children and Adults, Division of Pathology University-Hospital of Modena and Reggio Emilia, Modena, Italy; 11 Hemato-Oncology Program, CIMA, CCUN, University of Navarra, Pamplona, Spain; 12 CIBERonc, Instituto de Salud Carlos III, Madrid, Spain; 13 Departments of Oncology and Immunology, Clinica Universidad de Navarra, Pamplona, Spain; 14 Oncogenes and Effector Targets Laboratory, Solid Tumors Program, CIMA, CCUN, University of Navarra, Pamplona, Spain; 15 Molecular Therapeutics Program, CIMA, CCUN, University of Navarra, Pamplona, Spain; 16 Department of Gastroenterology and Hepatology, Navarra University Hospital Complex, Pamplona, Spain; 17 Hepatology Unit, Clínica Universidad de Navarra, CCUN, Pamplona, Spain; 18 Research Unit, Osakidetza Basque Health Service, Barrualde-Galdakao Integrated Health Organisation, Galdakao-Usansolo Hospital, Galdakao, Spain; 19 Department of Pathology, Navarra University Hospital Complex, Pamplona, Spain

**Keywords:** Pancreatic ductal adenocarcinoma, Epigenetic mechanisms, DNMT1, G9a, UHRF1, CM272, Apoptosis, Metabolic reprogramming, Chemotherapy sensitivity, Immunotherapy

## Abstract

**Background:**

Pancreatic ductal adenocarcinoma (PDAC) is a highly aggressive cancer with limited treatment options and a poor prognosis. The critical role of epigenetic alterations such as changes in DNA methylation, histones modifications, and chromatin remodeling, in pancreatic tumors progression is becoming increasingly recognized. Moreover, in PDAC these aberrant epigenetic mechanisms can also limit therapy efficacy. This study aimed to investigate the expression and prognostic significance of a key epigenetic complex encompassing DNA methyltransferase-1 (DNMT1), the histone methyltransferase G9a, and the scaffold protein UHRF1 in PDAC. We also evaluated the therapeutic potential of an innovative inhibitor targeting these epigenetic effectors.

**Methods:**

Immunohistochemical analysis of DNMT1, G9a, and UHRF1 expression was conducted in human PDAC tissue samples. Staining was semi-quantitatively scored, and overexpression was defined as moderate to strong positivity. The prognostic impact was assessed by correlating protein expression with patient survival. The antitumoral effects of the dual DNMT1-G9a inhibitor CM272 were tested in PDAC cell lines, followed by transcriptomic analyses to identify underlying mechanisms. The in vivo antitumoral efficacy of CM272 was evaluated in PDAC xenograft and syngeneic mouse models, both alone and in combination with anti-PD1 immunotherapy.

**Results:**

DNMT1, G9a, and UHRF1 were significantly overexpressed in PDAC cells and stroma compared to normal pancreatic tissues. Simultaneous overexpression of the three proteins was associated with significantly reduced survival in resected PDAC patients. CM272 exhibited potent antiproliferative activity in PDAC cell lines, inducing apoptosis and altering key metabolic and cell cycle-related genes. CM272 also enhanced chemotherapy sensitivity and significantly inhibited tumor growth in vivo without detectable toxicity. Combination of CM272 with anti-PD1 therapy further improved antitumor responses and immune cell infiltration, particularly CD4 + and CD8 + T cells.

**Conclusions:**

The combined overexpression of DNMT1, G9a, and UHRF1 in PDAC is a strong predictor of poor prognosis. CM272, by targeting this epigenetic complex, shows promising therapeutic potential by inducing apoptosis, reprogramming metabolic pathways, and enhancing immune responses. The combination of CM272 with immunotherapy offers a novel, effective treatment strategy for PDAC.

**Supplementary Information:**

The online version contains supplementary material available at 10.1186/s13046-024-03268-5.

## Background

Pancreatic ductal adenocarcinoma (PDAC) is among the most lethal cancers, currently ranking 7th in global cancer-related mortality and expected to become the 2nd leading cause of cancer deaths by 2030 [1, 2]. Despite significant research efforts, its occurrence continues to increase each year [1]. PDAC patients have a very poor prognosis, with a 5-year survival rate of only 12%, making early detection vital for improving outcomes [2, 3]. The likelihood of developing PDAC is affected by unchangeable risk factors such as age, ethnicity, type II diabetes, genetic cancer syndromes, chronic pancreatitis or the development of intraductal papillary mucinous neoplasms, as well as by modifiable lifestyle factors [2]. Over 80–90% of patients are diagnosed with either inoperable or metastatic disease or experience recurrence or metastasis after surgery, necessitating palliative care. Depending on the patient’s performance status, current standard chemotherapy protocols for PDAC include combination therapies like 5-fluorouracil, irinotecan, and oxaliplatin (FOLFIRINOX) or gemcitabine and nab-paclitaxel, or monotherapy with gemcitabine. However, despite progress, these treatments yield only modest improvements in overall survival (OS) presenting a significant risk of toxicity [3, 4]. So far, therapy with immune checkpoint inhibitors (ICIs) or in combination with chemotherapy has shown limited to no efficacy in treating PDAC [5]. Pembrolizumab (PD-1 inhibitor) is the only FDA-approved ICI in metastatic microsatellite instability-high (MSI-H) PDAC tumors. However, MSI-H status is present only in about 2% of patients [6]. Therefore, effective systemic therapies for PDAC patients are much needed.

Genetic alterations in PDAC have been very well characterized, with over 90% of PDAC cases harboring gain of function *KRAS* mutations [7]. Noteworthy is the occurrence of subsequent deletions or loss-of-function mutations in tumor suppressor genes such as *TP53*, *CDKN2A* and *SMAD4* which further exacerbate the disease [8]. Many other germline mutations have been identified, including *APC*, *ATM*, *BRCA1*, *BRCA2*, and *MLH1*, among others [9]. While the genetic bases of this tumor have been well appreciated, epigenetic alterations are increasingly recognized as relevant contributors to various stages of PDAC tumor development and malignancy [10–13]. Epigenetic mechanisms control gene expression patterns without altering the DNA sequence by modulating the accessibility of the transcriptional machinery to target genes, a process critical for cellular identity development. Changes in these mechanisms can contribute to tumor evolution by increasing cancer cell proliferation and metastasis through the silencing of tumor suppressor genes or the activation of oncogenes. Genetic, environmental, and tumor-intrinsic factors, such as the tumor microenvironment (TME), likely interact to create distinct epigenetic landscapes that contribute to PDAC heterogeneity [12]. The uniformity of driver gene mutations between primary tumors and metastatic sites in PDAC patients also underscores the importance of epigenetic reprogramming in clonal fitness and tumor evolution essential for PDAC expansion and metastatic spread [14–16]. Genome-wide analysis of PDAC samples has linked the evolution of malignant traits leading to distant metastasis to extensive epigenetic changes, including global reprogramming of histone H3K9 and DNA methylation within large heterochromatin domains [17]. Actually, abnormal DNA methylation and post-translational histone modifications are among the key epigenetic changes contributing to PDAC heterogeneity and progression [12, 18, 19]. Additionally, it has been observed how mutational alterations in oncogenes, such as *KRAS*, lead to downstream signaling events that stimulate cell growth in part by modulating histone and DNA modifying enzymes [18]. PDACs with mutations in chromatin modifiers like *ARID1A*, *KMT2C*, and *KMT2D* are more likely to develop aggressive squamoid/squamous morphology and metastasis [16]. Consequently, current efforts are focused on developing diagnostic and therapeutic strategies for PDAC based on the dysregulated epigenetic state of the tumor. A great advantage from a pharmacological standpoint is that epigenetic changes, unlike genetic mutations, are a reversible phenomenon. Thus, the development of the so-called “epi-drugs” that modify aberrant epigenetic states represents a real opportunity to overcome PDAC. In fact, various clinical trials are currently testing epigenetic inhibitors alone or in combination with standard-of-care chemotherapy or with immunotherapy [20]. While the outcomes of these trials are eagerly awaited, the dismal prognosis faced by most PDAC patients warrants the identification of novel and more effective therapeutic avenues.

In this work, we have examined the expression of the epigenetic complex formed by G9a, DNMT1 and UHRF1 in PDAC tissues, and experimentally evaluated the antitumoral efficacy of its pharmacological targeting. G9a (EHMT2) is an euchromatic histone-lysine methyltransferase that in specific situations physically interacts with DNA methyltransferase 1 (DNMT1) and the adaptor molecule, ubiquitin-like with PHD and RING finger domains 1 (UHRF1), contributing to DNA methylation, epigenetic tumor-suppressor gene silencing, and cancer cell proliferation [21–23]. We have found how the combined overexpression of these epigenetic effectors is significantly associated with PDAC patients’ survival. We also tested a potent, first-in-class, selective, and reversible dual small-molecule inhibitor, CM272 [23–27], targeting G9a and DNMT1 activity and also disrupting UHRF1 in PDAC cell lines. In this study, we demonstrate a remarkable antitumor potential of CM272 in PDAC cells and in clinically-relevant in vivo experimental models, sensitizing cancer cells to different chemotherapeutic agents, and showing a promising efficacy in combination with immune checkpoint inhibition.

## Methods

### Human PDAC specimen collection

This study was conducted on a first cohort of 42 patients diagnosed with PDAC and treated at the University Hospital of Modena, Italy, and a second cohort of 49 patients diagnosed and treated at Hospital Universitario de Navarra, Spain, from January 2000 to February 2019.

Patients with histologically proven PDAC, diagnosed at early/surgical stage without previous neoadyuvant therapy, with available formalin-fixed, paraffin-embedded (FFPE) histological samples were eligible for our analyses. Study population included patients either with ab initio surgically resected tumor. Patients who had received systemic therapies or radiotherapy prior to histological sample collection were excluded. Written informed consent was provided by all patients, and the study protocol was approved by the medical ethics committee of each hospital. University Hospital of Modena (Comitato Etico dell'Area Vasta Emilia Nord- Protocol Number: 0013043/19—Pratica 299/2019/OSS/AOUMO) and Clinical Research Ethical Committee of Navarra (Project 2015/120). All patients provided written informed consent.

### Cell culture and treatments

Human PDAC cell lines (MIA PaCa-2, PANC-1, Panc8902) and murine PDAC cell lines (DT6606 and PAN02) were maintained in Dulbecco's Modified Eagle Medium (DMEM) that was enriched with 10% heat-inactivated Fetal Bovine Serum (FBS), penicillin (100 U/mL), and streptomycin (100 mg/mL). Another set of human PDAC cell lines (ASPC-1, NP18, and HPAF-II) and murine PDAC cell lines (511,950, PDAC80, PM12167) were cultured in Roswell Park Memorial Institute (RPMI) 1640 medium, also supplemented with 10% heat-inactivated FBS, penicillin (100 U/mL), and streptomycin (100 mg/mL), all from Gibco-Life Technology (Waltham, MA, USA). The human Pancreatic Duct Epithelial Cell Line (H6c7) was cultured in Keratinocyte SFM, + EGF + bovine pituitary extract (Invitrogen) supplemented with 1 × antibiotic‐antimycotic (Gibco-Life Technology).

PDAC cancer cell lines (MIA PaCa-2, PANC-1, AsPC-1, HPAF-II) were acquired from the American Type Culture Collection (ATCC). NP18 and DT6606 were provided by Dr. Ruben Hernandez, PAN02 cells were provided by Dr Pedro Berraondo and Panc8902, 511,950, PDAC80, PM12167 and H6c7 cells were provided by Dr. Silvestre Vicent, all three researchers from CIMA-University of Navarra, Pamplona, Spain.

Treatment times and dosages in the experiments are specified throughout the manuscript, with controls receiving equivalent concentrations of dimethyl sulfoxide (DMSO) (always < 0.1% of the final volume). For cell viability assays, 2,000–4,000 cells were plated into each well of a 96-well plate with the appropriate culture media. After 24 h, the media/drug was added and cells were incubated for an additional 72 h. Following this period, 20 µL of CellTiter 96 Aqueous One Solution Cell Proliferation Assay (Promega, Madison, WI, USA) was introduced. After three hours, absorbance at 490 nm was measured using a plate reader. The drug concentration required to inhibit cell growth by 50% compared to the untreated control (GI_50_) was determined through curve fitting using GraphPad Prism-v10.2.0 software as previously described [23]. The combination index (CI) for UNC0642 and AZA (5-azacytidine) was calculated as detailed [25]. For apoptosis detection, cells were plated and treated following the same indications as for viability determinations, and Caspase-Glo® 3/7 Assay (Promega, Madison, WI, USA) was employed according to manufacture´s instructions.

Chemotherapeutic agents’ preparation: Irinotecan, 5-FU, oxaliplatin, leucovorin and cisplatin were obtained from Selleckchem (Houston, TX, USA). Gemcitabine, UNC0642 and AZA were from Sigma-Aldrich (St. Louis, MO, USA). The drugs were dissolved in DMSO or nuclease-free water and stored at − 80˚C. Drugs constituting FOLFIRINOX were combined in the following molar ratios analogous to those used in patients: 1.00 irinotecan, 80.95 5-FU, 0.80 oxaliplatin, 1.07 leucovorin. CM272 was produced by WuXi AppTech (Shanghai, China).

### Histones extraction

Histones were isolated as described [23]. In brief, cells were lysed using a buffer composed of 10 mM Tris–HCl (pH 7.4), 10 mM NaCl, and 3 mM MgCl_2_. After centrifugation at 2,500 rpm for 10 min at 4 °C, the supernatant was discarded, and the pellets were re-lysed in the same buffer with the addition of 0.5% NP40 on ice for 10 min with gentle agitation. The nuclei were then pelleted by centrifugation at 2,500 rpm for 10 min at 4 °C and resuspended in a solution of 5 mM MgCl_2_ and 0.8 M HCl. This suspension was incubated on ice for 30 min to facilitate histone extraction. Following incubation, the samples were centrifuged at 14,000 rpm for 10 min at 4 °C to remove debris, and the supernatants were transferred to a clean tube. To precipitate the histones, 50% trichloroacetic acid was added. The resulting pellets were washed with acetone, air-dried, and then resuspended in a solution of 100 mM Tris–HCl (pH 7.5), 1 mM EDTA, and 1% sodium dodecyl sulfate (SDS). The concentration of histones in the extract was determined using the BCA assay (Pierce Technologies, Rockford, IL) according to the manufacturer's instructions.

### Immunoprecipitation

For immunoprecipitation (IP) experiments, cells were lysed using an IP buffer containing protease and phosphatase inhibitors (20 mM Tris–HCl at pH 8, 137 mM NaCl, 1% NP-40, and 2 mM EDTA) at 4 °C for 30 min with continuous rotation. The cell lysates were clarified by centrifuging at 12,000 rpm for 20 min at 4 °C. Afterward, protein concentration was measured, and 800 µg of protein were pre-cleared by incubating with 25 µl of Dynabeads G (Invitrogen) for 2 h with constant rotation at 4 °C. Simultaneously, 5 µg of the primary antibody and an equivalent amount of control IgG were incubated with 20 µl of Dynabeads G for 2 h at room temperature with continuous rotation. The antibody-bound Dynabeads were washed three times with citrate phosphate buffer (pH 5) containing 0.01% Tween-20 and then incubated overnight at 4 °C with the pre-cleared protein samples under constant rotation. The next day, the samples were washed three times using PBS (Gibco-Life Technology) with protease and phosphatase inhibitors, then boiled in Laemmli buffer at 95 °C for 5 min. The beads were separated using a magnetic rack, and the supernatant was collected for Western blot analysis. Antibodies used for IP were: G9a (435,200, Invitrogen) and IgG mouse (SC2025, Santa Cruz Biotechnology, Dallas, TX, USA).

### Immunoblot analyses

Cells were lysed in RIPA buffer as described previously [28]. Histone extracts, as well as IP products and homogenates from cells, were subjected to immunoblot (Western blot) analysis as reported [23]. Total protein was separated via sodium dodecyl sulfate polyacrylamide gel electrophoresis (SDS-PAGE), and electrotransferred onto PVDF membranes (Millipore, Burlington, MA, USA). The following primary antibodies were used for protein detection: anti-H3K9me2 (1:1000, 07–212, Millipore), anti-Total H3 (1:2000, 05–928, Millipore), anti-ATF3 (1:1000, sc-188, Santa Cruz Biotechnology), anti-CDK1A (1:1000, ab188224, Abcam, Cambridge, UK), anti-EGFR (1:1000, 06–847, Millipore), anti-alpha-TUBULIN (1:1000, 2144, Cell Signaling, Danvers, MA, USA), anti-G9a (1:1000, 33,065, Cell Signaling), anti-DNMT1 (1:1000 5032, Cell Signaling) and anti-UHRF1 (1:500, ab57083, Abcam). Secondary HRP-conjugated goat anti-mouse IgG (1:5000, 68,860, Cell Signaling) or anti-rabbit IgG (1:5000, 2729, Cell Signaling) were also used. Target antigens were visualized using SuperSignal™ West Pico PLUS chemiluminescent substrate (Thermo Fisher Scientific, Waltham, MA, USA). Images were scanned with a ChemiDoc Imaging System (Bio-Rad, Hercules, CA, USA).

### 5meC Immunofluorescence

For immunofluorescence staining, cells were grown on coverslips and treated with either vehicle or CM272 for 72 h. They were then fixed using ice-cold methanol for 15 min at room temperature and subsequently washed twice with PBS. To quench, cells were exposed to 50 mM NH_4_Cl in PBS for 10 min. Following three washes with PBS, cells were permeabilized with 0.2% Triton X-100 for 5 min at 4 °C, and DNA was denatured using 4 M HCl for 15 min, followed by treatment with 100 mM Tris–HCl (pH 8.5) for 10 min. After washing, coverslips were blocked with Superblocking buffer (Thermo Fisher Scientific) for 1 h at room temperature. They were then incubated overnight at 4 °C with anti-5-methylCytosine antibody (Eurogentec, Seraing, Belgium, BI-MECY 0100), diluted in 1% BSA in PBS. After washing, cells were treated with fluorophore-conjugated secondary antibodies in 1% BSA in PBS for 1 h at room temperature, washed again, and then stained with Vectashield (Vector Laboratories, Burlingame, CA, USA) containing DAPI. Images were captured using a Zeiss Axio Imager.M1 microscope (Zeiss, Oberkochen, Germany).

### Colony formation assays

Colony formation assays were conducted using MIA PaCa-2, PANC-1, AsPC-1, and NP18 cells following the protocol outlined previously [23]. In brief, 3,000 cells were plated in complete medium in six-well plates and treated with CM-272 the following day. The medium was replaced every two days, and cultures were maintained until observable differences between treatment conditions emerged (approximately 4 weeks). After the incubation period, plates were washed with PBS, fixed with 4% formaldehyde (Sigma) in PBS for 10 min, and stained with crystal violet. Representative images were captured.

### siRNAs

Human-specific siRNAs targeting G9a, DNMT1, and UHRF1, along with control siRNA (siC), were sourced from Santa Cruz Biotechnology (Santa Cruz, CA). Transfections were carried out using 75 nM of each siRNA with Lipofectamine RNAiMAX reagent (Invitrogen, Grand Island, NY, USA), following the protocol detailed previously [28] and according to the manufacturer's guidelines. When multiple siRNAs were used simultaneously, each specific siRNA was applied at 32.5 nM for combinations of two siRNAs or 25 nM for combinations of three siRNAs. Cells were collected 48 h post-silencing, and gene expression was assessed by qPCR following the transfections.

### RNA isolation and quantitative real-time RT-qPCR

Total RNA from cell lines was extracted using the automated Maxwell system from Promega (Madison, WI, USA). For reverse transcription, RNA samples were initially heated at 90 °C for 1 min to denature, followed by incubation at 37 °C for 1 h. The reverse transcription mix contained 50 mM Tris–HCl (pH 8.3), 75 mM KCl, 3 mM MgCl_2_, 10 ng/μL of random primers, 0.5 mM of each deoxyribonucleic triphosphate (dNTP), 5 mM dithiothreitol (DTT), 1.2 U/μL RNase inhibitors (RNase Out), and 6 U/μL M-MLV reverse transcriptase enzyme. All reagents were sourced from Invitrogen (Carlsbad, CA, USA), except for dNTPs, which were obtained from Roche Diagnostics (Mannheim, Germany). Quantitative reverse transcription PCR (qRT-PCR) was conducted as described, with gene expression levels normalized to the housekeeping gene H3F3A as previously outlined [23]. Primer sequences are available upon request.

### RNA sequencing (RNAseq)

RNA was assessed for quantity and quality using the Qubit HS RNA Assay Kit (Thermo Fisher Scientific) and the 4200 Tapestation with High Sensitivity RNA ScreenTape (Agilent Technologies, Santa Clara, CA, USA). All RNA samples met high-quality standards, with RIN values above 8. Library preparation was carried out with the Illumina Stranded mRNA Prep Ligation kit (Illumina, San Diego, CA, USA) according to the manufacturer’s instructions. Sequencing libraries were prepared from 100 ng of total RNA. The process involved selecting and purifying poly(A)-containing RNA molecules with magnetic beads coated with poly(T) oligos. These poly(A)-RNAs were then fragmented and reverse transcribed into the first cDNA strand using random primers. The second cDNA strand was synthesized with dUTP to maintain strand specificity. The resulting cDNA fragments were purified with AMPure XP beads (Beckman Coulter, Brea, CA, USA), adenylated at the 3′ ends, and ligated with uniquely indexed sequencing adapters. After purification, the fragments were PCR amplified to generate the final libraries. The quality and quantity of these libraries were confirmed using the Qubit dsDNA HS Assay Kit (Thermo Fisher Scientific) and the 4200 Tapestation with High Sensitivity D1000 ScreenTape (Agilent Technologies). The libraries were sequenced on a NextSeq2000 sequencer (Illumina), yielding 30–40 million paired-end reads per sample. The data were demultiplexed using Cutadapt. RNAseq was performed at the Genomics Unit of the Center for Applied Medical Research (CIMA), Universidad de Navarra, Pamplona, Spain.

### Immunohistochemical analyses

Three-micrometer-thick sections were prepared from formalin-fixed paraffin-embedded pancreatic tissues. The sections were deparaffinized using xylene, dehydrated with ethanol, and treated with 3% hydrogen peroxide to inhibit endogenous peroxidase activity. Antigen retrieval was achieved by heating the sections in a 10 mM Tris–EDTA buffer at pH 9 before incubating with primary antibodies for G9a (1:200, ab185050, Abcam), DNMT1 (1:100, ab188453, Abcam), UHRF1 (1:100, ab194236, Abcam), CD8 (1:100, 98941 T, Cell Signalling), CD4 (1:100, ab183685, Abcam), CDKN1A (1:100, ab188224, Abcam), and anti-H3K9me2 (1:100, 07–212, Millipore). For detection, an HRP-conjugated Envision secondary antibody (K4003, Dako, Santa Clara, CA, USA) was used, followed by DAB reagent (K3468, Dako). Signal quantification was performed using QuPath software v0.3.217. The tissue sections were counterstained with Hematoxylin (Sigma-Aldrich) and dehydrated. Negative controls were included by omitting the primary antibodies.

### In vivo experiments

For the patient-derived xenograft (PDX) model, experiments performed at Vall d'Hebron Institute of Oncology (Barcelona, Spain) were approved by the institution's Ethical Committee and the Catalan Regional Government. PDX92 was created by implanting 3–4 mm tumor fragments from a metastatic liver biopsy of a PDAC patient into the flanks of 6-week-old female NOD.CB-17-Prkdc scid/Rj mice (Janvier Labs, Saint-Berthevin, France, RRID:MGI:3,760,616). When tumors reached 100–150 mm^3^, mice were divided into two groups for daily intraperitoneal treatment (i.p.) with either vehicle (PBS) or CM272 (5 mg/kg) for 28 days. Tumor growth and animal weight were monitored bi-weekly. Mice were euthanized when tumors reached 1–1.5 cm^3^ or if significant weight loss occurred. Mice were kept in filtered cages with a 12-h light/dark cycle and had ad libitum access to food and water.

For the orthotopic PDAC model, 8-week-old male C57/BL6 mice (Jackson Laboratories, Bar Harbor, ME, USA) were housed four per cage with standard chow and water at 22 °C on a 12-h light/dark cycle. A 1.5-cm midline laparotomy was performed under anesthesia, and DT6606 cells (5 × 10^5^ cells) were injected into the pancreas. The abdominal wall was closed with 5–0 Vicryl suture (Ethicon, Raritan, NJ, USA). Mice were randomly assigned to two different treatment groups: (1) saline, (2) CM272 (5 mg/kg, i.p., 5 times/week). After 4 weeks, mice were euthanized, and tumors were excised, weighed, and processed for histology and immunohistochemistry.

In a combination treatment model, 1 × 10^6^ PAN02 cells were injected subcutaneously into the right flank of 8-week-old male C57/BL6 mice. When tumors reached ~ 100 mm^3^, mice (*n* = 6) were divided into four treatment groups: (1) saline; (2) CM272 (5 mg/kg, i.p., 5 times/week); (3) anti-PD1 (10 mg/kg, i.p., twice a week); and (4) combination (CM272 and anti-PD1, using the same dosis and times). After 4 weeks, mice were euthanized, tumors excised, weighed, and analyzed for histology and immunohistochemistry. These procedures were also approved by the University of Navarra's Animal Care Committee (ethical committee approval #R-CP001-15GN).

### Biochemical parameters

Serum levels of alanine aminotransferase (ALT), aspartate aminotransferase (AST), lipase (LIPC), and amylase (AMYL) were measured using a C311 Cobas Analyzer (Roche Diagnostics GmbH, Mannheim, Germany) following manufacturer’s instructions.

### Statistical analyses

Association between IHC expression of DNMT1, G9a and UHRF1 was tested using Fisher exact test. Disease-free survival (DFS), overall survival (OS) and their two-sided 95% CI were estimated by the Kaplan–Meier method and curves were compared by the log-rank test (at a significance level of 5%). Estimated HRs and their two-sided 95% CI were calculated using the Cox-proportional hazard model. Other statistical analyses were conducted using GraphPad Prism 10.2.0 software. Comparisons between two groups were made using either the paired two-tailed Student’s t-test or the Kruskal–Wallis ANOVA test, depending on the data distribution. Survival rates were evaluated with Kaplan–Meier curves, and differences were tested using the log-rank test. All *p*-values were two-tailed, and significance was defined as *p* < 0.05.

## Results

### Analysis of DNMT1, G9a, and UHRF1 expression in PDAC

**Fig. 1:**
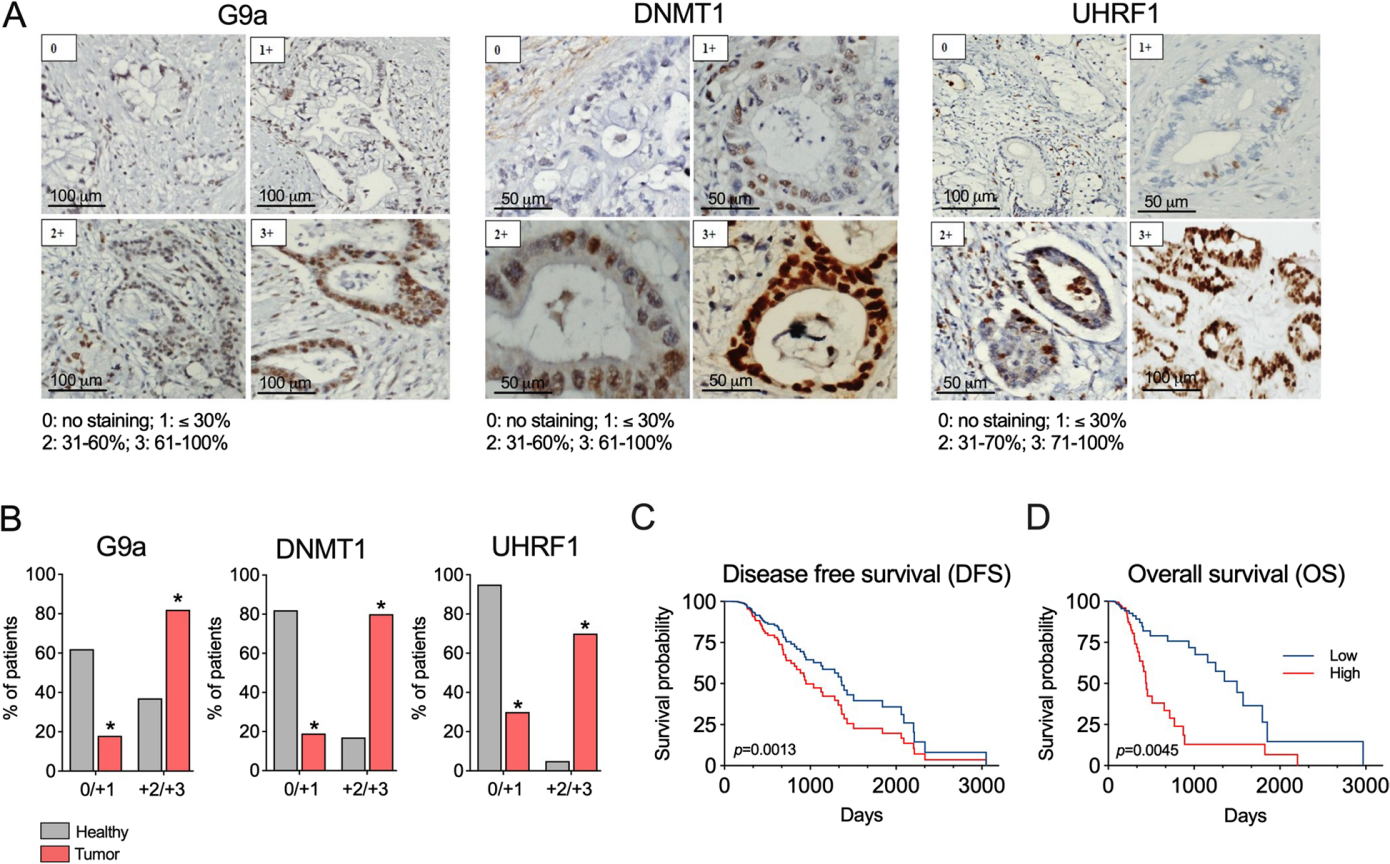
Expression of G9a, DNMT1, and UHRF1 mRNA in human PDAC*.* **A** Scoring of G9a, DNMT1 and UHRF1 expression in pancreatic tissues from PDAC patients. Each image shows the representative IHC staining of the three proteins according to each score assigned. **B** Differential G9a, DNMT1 and UHRF1 expression (3 +) versus (2 + /1 + /0) in tumor tissue and normal pancreatic ducts. **C** DFS and (**D**) OS in PDAC patients according a combined high expression (3 +) or low expression (2 + /1 + /0) of DNMT1/G9a/UHRF1. *p < *0.05*

Immunohistochemical (IHC) staining for DNMT1, G9a, and UHRF1 was performed in an initial cohort of 42 PDAC patients treated at Modena University Hospital, Italy (Suppl Table 1). All PDAC tissues expressed G9a and UHRF1, and most (73.8%) were positive for DNMT1. The expression of all three proteins was confined to the nucleus, with no staining observed in the cytoplasm or plasma membrane, in agreement with previous reports [25, 29–31]. Using a semi-quantitative scoring system (Fig. 1A), most cases showed strong positivity for G9a (88%) and UHRF1 (76,1%), indicated as score 3 + . The intensity levels of DNMT1 staining varied among samples (Suppl Table 2). Whenever possible, the staining of other tissue components (stroma, normal ducts, PanIN) within histological specimens was also evaluated. DNMT1-negative stroma was reported in 6% (2/36) of patients. Most cases showed weak (58%, 21/36) or moderate (22%, 8/36) DNMT1 positivity in stroma, with only 14% (5/36) showing score 3 + stroma. G9a staining in stroma was weak (1 +) or moderate (2 +) in 40.5% (15/37) and 46% (17/37) of cases, respectively, with 13.5% (5/37) scoring 3 + . For UHRF1, stroma was 1 + or 2 + in 58.5% (24/41) and 34% (14/41) of cases, respectively, with 7.5% (3/41) scoring 3 + . Normal pancreatic ducts mostly stained either negative or weakly positive for the three proteins. To assess the differential expression of DNMT1, G9a, and UHRF1 in tumor tissue and normal pancreatic ducts, we compared the highest level of expression (score 3 +) versus lower levels (score 0, 1 + , and 2 +). G9a and UHRF1 were significantly overexpressed in cancer compared to normal ducts (*p* < 0.001 for both), although DNMT1 was not significantly overexpressed in this comparison (Suppl Table 3).

To validate these results and give robustness to our analysis, we expanded the number of cases performing similar analyses in a different cohort including 49 PDAC patients from the University Hospital of Navarra, Spain (Clinical characteristics described in Suppl Table 1). Here, we directly analyzed the expression of the three proteins in tumor and normal pancreatic ducts comparing the highest level of expression (score 3 +) versus lower levels (score 2 + , 1 + , and 0) (Suppl Table 3). When both cohorts were analyzed together, we confirmed the prevalence of higher DNMT1, G9a, and UHRF1 immunostaining scores (score 3 +) in cancer cells compared to normal ducts, in which lower levels (score 0, 1 + , and 2 +) were more prevalent. (Fig. 1B, Suppl Table 3).

### Correlation of DNMT1, G9a, and UHRF1 expression with clinicopathological variables

The expression of DNMT1, G9a, and UHRF1 (scores 3 + versus 2 + , 1 + and 0) in tumor was correlated with various clinicopathological variables: sex, tumor site (pancreatic head vs. body/tail), pT stage (pT1, pT2, pT3), pN stage (pN0, pN1, pN2), vascular invasion, perineural invasion, tumor grade (G3 vs. G1/G2) and need of adjuvant therapy. Patients who had a more advanced tumor stage (pT) more frequently exhibited an overexpression of DNMT1 and G9a. Similarly, patients with greater lymph node involvement (pN) showed an overexpression of DNMT1 and UHRF1. Additionally, microvascular invasion in the surgical specimen was associated with an overexpression of G9a and UHRF1, and perineural invasion was associated with the overexpression of G9a (Suppl Table 4). Interestingly, analyzing the three epigenetic modifiers together, we found that the simultaneous overexpression (3 +) of at least two of the three proteins (DNMT1, G9a, and UHRF1) in cancer cells resulted in a significant shorter DFS and OS when all PDAC patients were analyzed together (Fig. 1C).

These results corroborate the overexpression of the three proteins in PDAC tumors and demonstrate that, although the independent overexpression of these three epigenetic modifiers can be associated with specific clinicopathological characteristics in PDAC patients, the simultaneous elevation of G9a, DNMT1, and UHRF1 levels in tumor tissues is linked to a significantly poor prognosis.

### Expression and protumorigenic role of G9a, DNMT1 and UHRF1 in PDAC cells

**Fig. 2:**
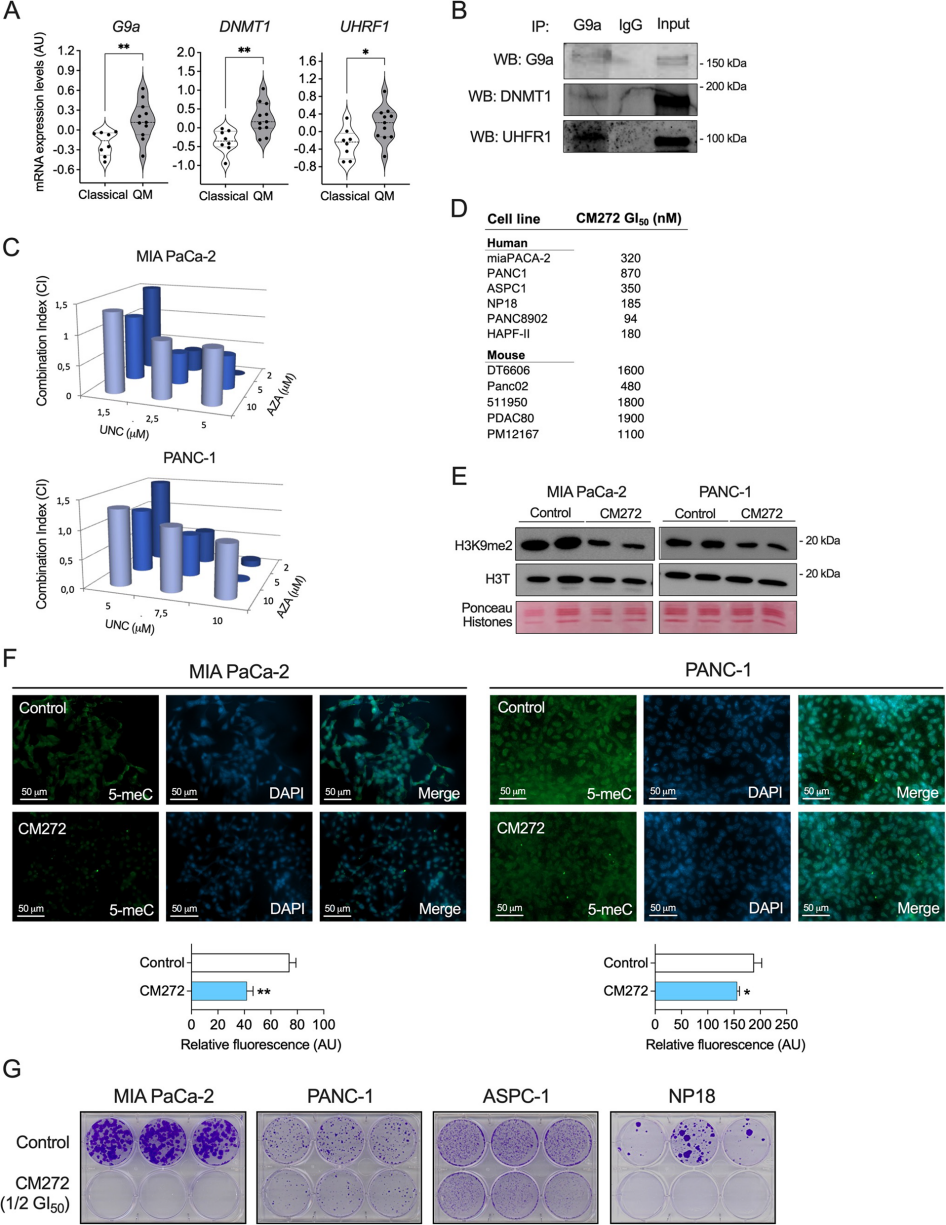
Expression and simultaneous targeting of G9a, DNMT1 and UHRF1 in human PDAC cell lines. **A** G9a, DNMT1 and UHRF1 mRNA levels in PDAC cell lines according to their classification as classical (*n* = 9) or quasimesenchymal, QM (basal) (*n* = 36) categories extracted from Collisson dataset. **B** Immunoprecipitation of endogenous G9a in MIA PaCa-2 cells. Immunoprecipitates were probed with anti-G9a, anti-DNMT1 and anti-UHRF1 antibodies. Pre-immune IgG immunoprecipitates and Inputs are shown as controls. **C** Combination study of the growth inhibitory effects of G9a (UNC0642, UNC) and DNMT1 (AZA) inhibitors in MIA PaCa-2 and PANC-1 cells. **D** GI_50_ values for CM272 in the indicated human and mouse PDAC cell lines. **E** Effect of CM272 treatment (72 h) on the levels of H3K9me2 in MIA PaCa-2 and PANC-1 cells. H3 total (H3T) levels and isolated histones (Ponceau) are also shown. **F** Effect of CM272 treatment (72 h) on the global levels of DNA CpG methylation in MIA PaCa-2 and PANC-1 cells. **G** Colony formation assays in MIA PaCa-2 and PANC-1 cells treated with CM272 at half of their respective GI_50_. *p < 0.05; **p < *0.01*

To further evaluate the relevance of this epigenetic complex in PDAC, we examined *G9a*, *DNMT1*, and *UHRF1* mRNA expression levels in publicly available datasets obtained from a broad panel of human PDAC cell lines [32]. Interestingly, significantly higher expression levels of the three epigenetic effectors were found in PDAC cell lines belonging to the “basal” subtype compared with those conforming the “classical” group (Fig. 2A). These data align with previous observations, where specific epigenetic events consistently distinguished these two major PDAC subtypes, the basal-like type associated with a more mesenchymal gene expression profile, higher tumor grade, chemoresistance, and poor prognosis; and the classical subtype, characterized by an epithelial-like gene signature, lower tumor grade, and better prognosis [7, 12, 32]. Through co-immunoprecipitation assays, we observed that these three epigenetic modifiers indeed appeared in the same complex in MIA PaCa-2 cells (Fig. 2B). To demonstrate that the simultaneous inhibition of the G9a/DNMT1/UHRF epigenetic complex could be an improved strategy to quell PDAC cells growth, we treated MIA PaCa-2 and PANC-1 cells with the G9a specific inhibitor UNC0642 and the DNMT inhibitor 5’-azacytidine (AZA) simultaneously at different concentrations. Initially, the single administration of each epigenetic inhibitor resulted in relatively high GI_50_ values for both cell lines (in the micromolar range for UNC0642 and in the millimolar range for AZA) (Fig. S1A). However, the combined treatment showed a synergistic growth inhibitory effect in both PDAC cell lines at several concentrations tested (Fig. 2C). We then evaluated the effect of CM272, our lead G9a/DNMT/UHRF1 inhibitory compound, on PDAC cell viability. We observed very potent effects, with GI_50_ values in the nM range for most human and mouse PDAC cell lines tested (Fig. 2D). Interestingly, we found a significantly superior GI_50_ value when we tested H6c7 cells, an epithelial non tumoral pancreatic cell line (Fig. S1B).

Next, we evaluated the on-target effects resulting from the inhibition of G9a and DNMT1 epigenetic activities. As expected, CM272 decreased the total cellular contents of H3K9me2 (Fig. 2E) and DNA methylation (5-methyl-cytosine [5meC]) levels (Fig. 2F). Noteworthy, CM272 markedly impaired the clonogenic capacity of PDAC cells even when tested at half its GI_50_ concentrations (Fig. 2G). Taken together, these observations indicate that the epigenetic inhibitor CM272 is very effective against PDAC cells.

### Mechanisms of CM272 antitumoral activity in PDAC cells

**Fig. 3:**
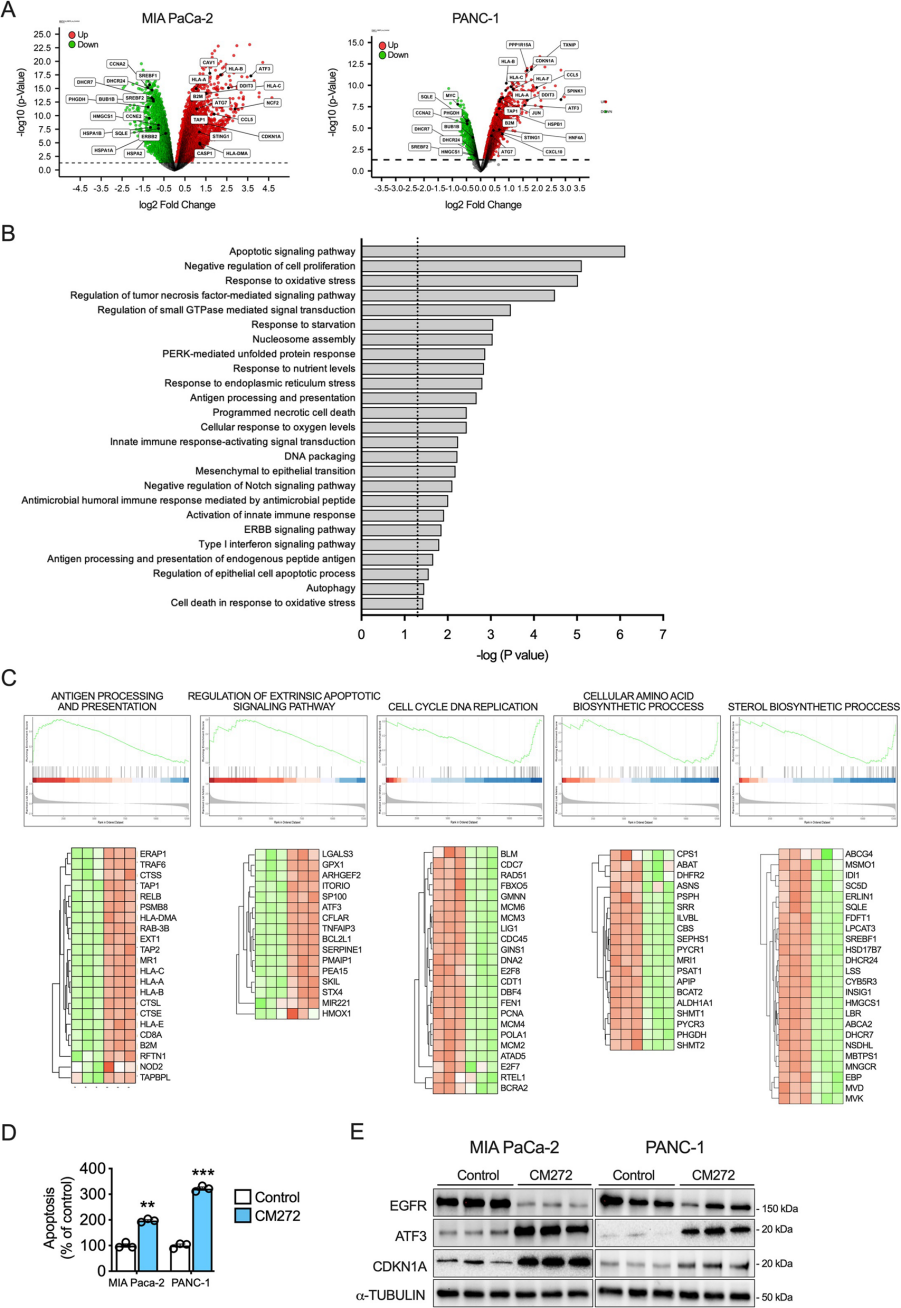
Mechanisms of CM272 antitumoral activity in PDAC cells. **A** Volcano plot of the genes differentially expressed in MIA PaCa-2 (left panel) and PANC-1 (right panel) cells treated with CM272 (GI_50_, 72 h). Selected functionally relevant genes are indicated. **B** Most relevant GO functional categories of genes undergoing changes in expression identified by RNA-sequencing in MIA PaCa-2 cells treated with CM272 (GI_50_, 72 h). **C** GSEA analysis of specific categories including heatmaps with a ranked list of genes modulated by CM272 from the RNA-seq data. **D** Apoptosis *vs* Viability determination in PDAC cells treated with vehicle (Control) or CM272 at their GI_50_ during 72 h. **E** Representative western blots of the indicated proteins in Control and CM-272-treated MIA PaCa-2 and PANC-1 cells. Blots were probed for α-TUBULIN to show equivalent loading. **p < 0.01; ***p < *0.001*

To characterize the molecular mechanisms that underlie the antitumoral effects of CM272, we performed transcriptomic studies in control and CM272-treated MIA PaCa-2 and PANC-1 PDAC cells. In the case of MIA PaCa-2 we detected 5048 upregulated and 3431 downregulated genes compared with controls (*p* < 0.01) from 12,655 genes analyzed, while PANC-1 cells showed 3050 upregulated and 2102 downregulated genes after CM272 treatment (*p* < 0.01) from 10,266 genes analyzed. (Fig. 3A). Gene ontology (GO) functional classification of differentially expressed genes revealed general categories that were very conserved between the two PDAC cell lines (Fig. 3B and Fig. S2A). Many of the processes identified were linked to apoptosis signaling pathways, oxidative stress, endoplasmic reticulum stress and unfolded protein responses, and to the negative regulation of intracellular signal transduction pathways, such as ERK1/2 cascade and ERBB signaling. (Fig. 3B). These observations are consistent with previous transcriptomic analyses in which *G9a* was genetically depleted in PDAC models [33]. Interestingly, antigen processing and presentation via major histocompatibility complex (MHC) Class I, type I interferon signaling, and processes related to T cell mediated immunity were among the most enriched categories elicited by CM272 treatment in both cell lines. Besides “antigen presentation” and “apoptosis-related” pathways, more detailed scrutiny of transcriptomic data using gene-set enrichment analysis (GSEA) also revealed additional categories linked to the inhibition of cell cycle, amino acid metabolism and cholesterol biosynthetic processes (Fig. 3C and Fig. S2B).

In agreement with transcriptomic analyses, we found that treatment of MIA PaCa-2 and PANC-1 cells with CM272 resulted in the upregulation of important immune response genes, such as those belonging to the HLA family or *TAP1* and *B2M*, key components of the antigen presentation machinery responsible for neoantigen presentation to CD8 + and CD4 + T cells [34]. Similarly, the expression of the transcription factor *ATF3* and that of *BCL2L1*, involved in the cellular stress response and cell death respectively, were also induced (Fig. 3C and Fig. S2B). Important genes required for progression through the cell cycle, such as *E2F8*, *PCNA* or *MCM2* were downregulated after CM272 treatment. Also consistent with our transcriptional analyses, the expression of genes implicated in sterol biosynthetic processes such as *SQLE*, *HMGCS1* and *DHCR24*, or those involved in amino acid biosynthetic process, such as *PYCR1*, *BCAT2* or *PHGDH* were significantly repressed.

We validated some of the key transcriptomic effects of G9a/DNMT1/UHRF1 inhibition by demonstrating increased levels of apoptosis in PDAC cells treated with CM272 (Fig. 3D), as well as by confirming the upregulation of ATF3 and CDKN1A-p21 proteins, involved in oxidative stress response and cellular senescence, respectively (Fig. 3E). Interestingly, we also observed decreased levels of epidermal growth factor receptor (EGFR or ERBB1) protein (Fig. 3E). The dysregulation of these proteins is known to play very relevant roles in PDAC development [33, 35–37].

### CM272 enhances the response of PDAC cells to chemotherapy

**Fig. 4:**
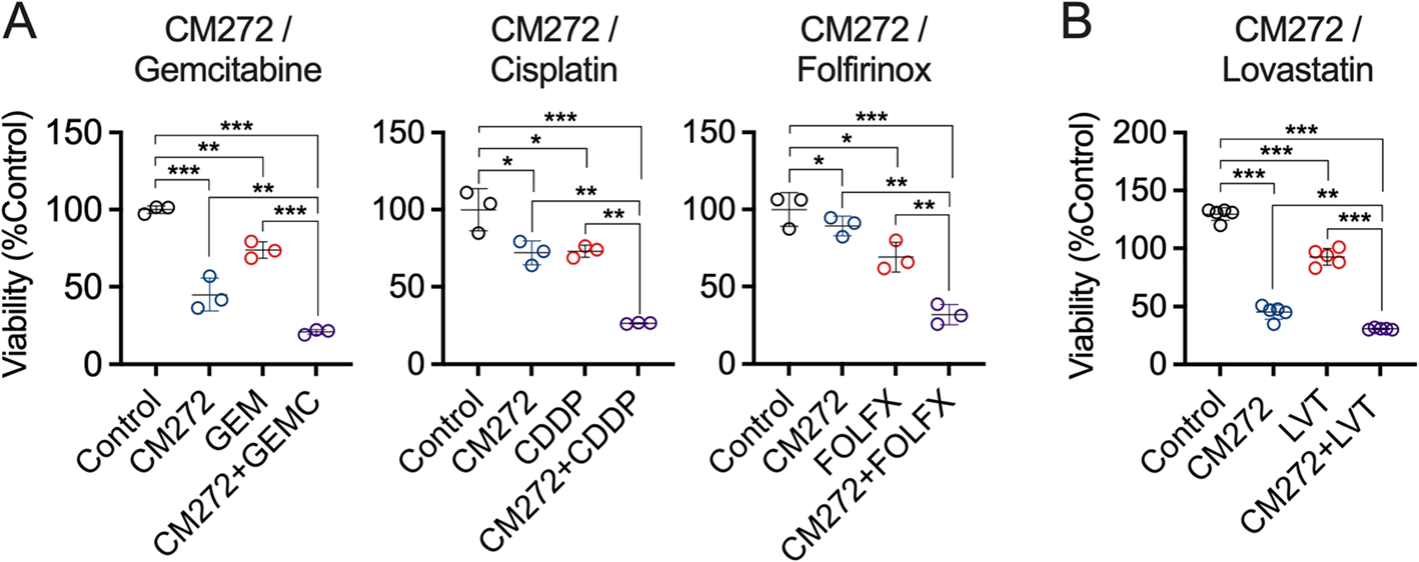
CM272 synergizes with antitumor drugs in the inhibition of PDAC cells growth. **A** Combination study of the growth inhibitory effects of CM272 and gemcitabine (GEM), cisplatin (CDDP) and FOLFIRINOX (FOLFX) in MIA PaCa-2 cells. **B** Combination study of the growth inhibitory effects of CM272 and lovastatin (LVT) in MIA PaCa-2 cells. *p < 0.05; **p < 0.01; ***p < *0.001*

Epigenetic mechanisms alterations appear to play a pivotal role not only in cancer initiation and progression, but also in chemoresistance. This evidence is paving the way for new treatment strategies, involving the use of epi-drugs in combination with conventional anti-cancer therapies [38, 39]. In view of this, we wanted to evaluate whether CM272 was able to increase the susceptibility of PDAC cells to chemotherapeutic agents currently used in PDAC patients. To test this notion, we treated MIA PaCa-2 cells with CM272 and gemcitabine and found that such combination therapy resulted in a more pronounced decrease in cell viability than did either drug alone. Moreover, we observed a similar effect in cells treated with CM272 in combination with cisplatin, and also in PDAC cells treated with CM272 together with FOLFIRINOX, a combination of 5-fluorouracil (5-FU), oxaliplatin (Oxa), irinotecan and leucovorin [40] (Fig. 4A). Together, our findings demonstrate that the pharmacological inhibition of the G9a/DNMT1/UHRF1 complex enhances the cytotoxic response to conventional chemotherapeutic agents in pancreatic cancer cells.

According to our transcriptomic analyses, one pathway that was markedly inhibited in PDAC cells upon CM272 treatment was the sterol biosynthetic process (Fig. 3C). Metabolic reprogramming is a hallmark of cancer cells [41], and among various metabolic pathways, alterations in cholesterol metabolism have been related to PDAC development [42]. Cholesterol is an essential molecule for membranes formation and cell proliferation, and cancer cells need to synthesize large amounts of cholesterol to meet the energetic demands associated with rapid growth [43, 44]. In this context, treatment of PDAC cells with lovastatin, an inhibitor of 3-hydroxy-3-methylglutaryl-CoA (HMG-CoA) reductase, the rate-limiting enzyme in cholesterol synthesis, has been demonstrated to promote apoptosis [45]. This knowledge led us to evaluate if CM272 treatment could also enhance the effect of lovastatin on the viability of PDAC cells. As shown in Fig. 4B, we observed that the inhibitory effects of lovastatin on MIA PaCa-2 cells growth was significantly enhanced by the concomitant treatment with CM272. Interestingly, we confirmed the specificity of this effect on PDAC tumor cells, as when we performed these treatments combining CM272 with chemotherapeutic agents on the H6c7 cell line, we did not observe any further decrease in viability or increase in apoptosis. (Fig S3).

### Inhibition of the G9a/DNMT1/UHRF1 complex potentiates the immunogenicity of PDAC cells

**Fig. 5:**
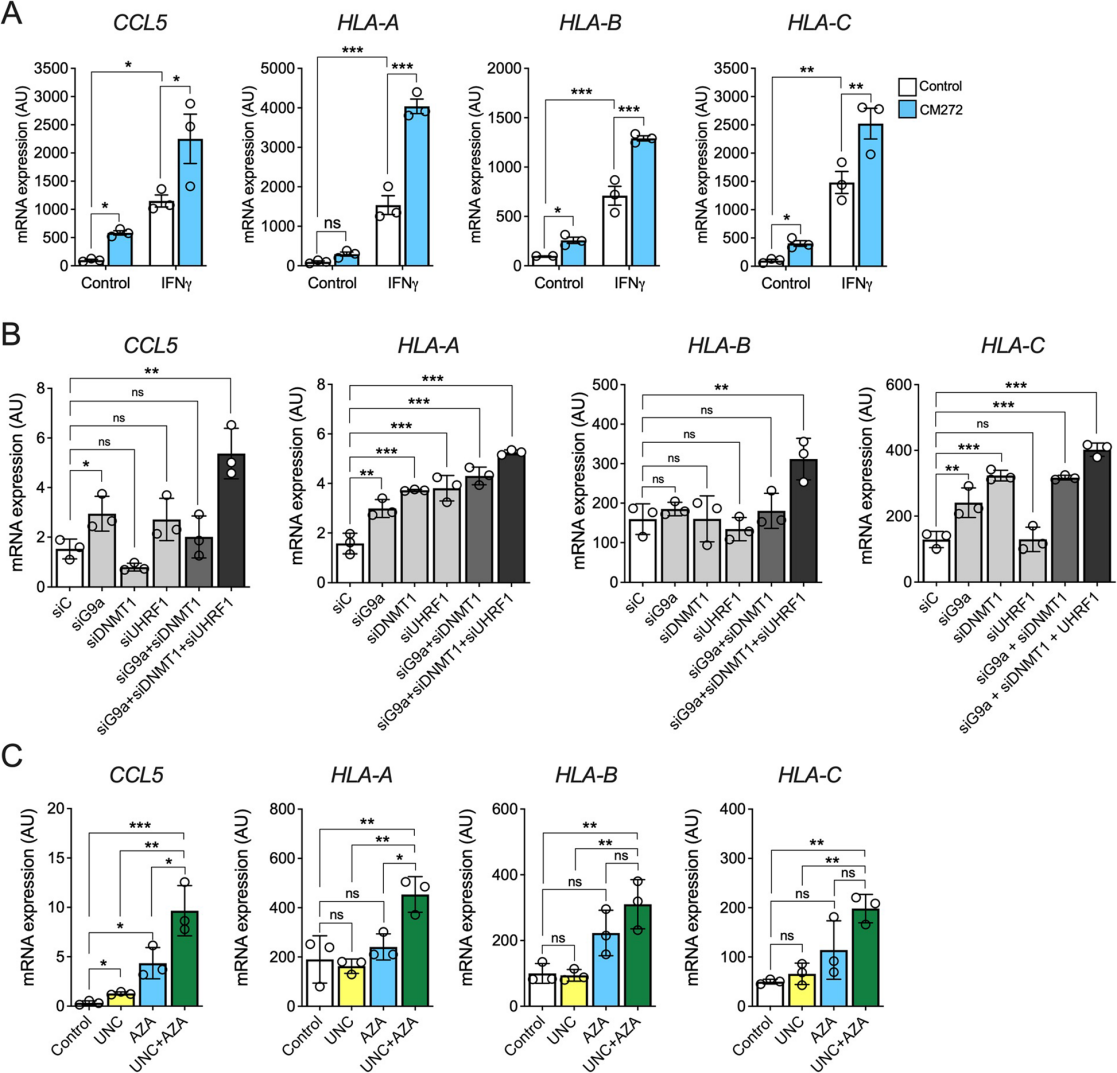
The simultaneous inhibition of the epigenetic complex G9a/DNMT1/UHRF1 activates the expression of immune response-related genes in PDAC cells. **A** qPCR analysis of *CCL5, HLA-A, HLA-B* and *HLA-C* expression in MIA PaCa-2 cells pretreated with CM-272 for 24 h and then induced with IFNγ (100U/mL) for another 24 h. **B** qPCR analysis of *CCL5, HLA-A, HLA-B* and *HLA-C* expression in MIA PaCa-2 cells transfected with G9a, DNMT1 or UHRF1-specific siRNAs, specific siRNAs combinations (siG9a + siDNMT1 and siG9a + SiDNMT1 + siUHRF1) and control siRNAs (siC) for 48 h. **C** qPCR analysis of *CCL5, HLA-A, HLA-B* and *HLA-C* expression in MIA PaCa-2 cells treated with UNC, AZA, and the combination (UNC + AZA) at their GI_50_ during 72 h. *p < 0.05; **p < 0.01; ***p < *0.001;* ns*: not significant*

In our GSEA of the transcriptome of CM272-treated PDAC cells “antigen processing and presentation” was one of the pathways most significantly upregulated (Fig. 3C). Given the potential relevance of this response regarding tumor immunogenicity, and eventually for leveraging the efficacy of ICI-based therapies, we validated the effect of CM272 on the expression of chemokine *CCL5* and different MHC genes such as *HLA-A, HLA-B* and *HLA-C* (Fig. 5A and Fig. S4A). Remarkably, pre-incubation of PDAC cells with CM272 significantly enhanced the response of these and other immune response-related genes such as *CXCL10*, *TAP1* and *B2M*, to IFNγ, the key immune effector cytokine (Fig. 5A and Fig. S4A and S4B). We also examined in more detail the contribution of each component of the G9a/DNMT1/UHRF1 epigenetic complex in this response. To this end, we used specific siRNAs to knockdown the expression of *G9a*, *DNMT1* and *UHRF1* individually or in combination. Interestingly, the simultaneous inhibition of the three components (Fig. S5A) caused the highest induction in the mRNA levels of the chemokine *CCL5* and the HLA genes *HLA-A, HLA-B* and *HLA-C* (Fig. 5B and Fig. S5B). These results were pharmacologically validated when MIA PaCa-2 cells were treated with specific inhibitors of G9a (UNC0642) and DNMT1 (AZA), with the combined treatments resulting in the highest induction of the expression of these immune response-related genes (Fig. 5C and Fig. S6). All these data align with previous evidence showing how cancer cells downregulate immune sensing via epigenetic silencing of antitumor cytokines, chemokines, or components of Class I MHC [11, 46]. In view of our findings the G9a/DNMT1/UHRF1 complex would play such a key role in PDAC cells. Therefore, these results strongly argue for a therapeutic strategy in which the simultaneous inhibition of the epigenetic complex G9a/DNMT1/UHRF1 could be leveraged to prime the PDAC tumor and its microenvironment for a better response to immune therapies.

### In vivo antitumor activity of CM272

**Fig. 6:**
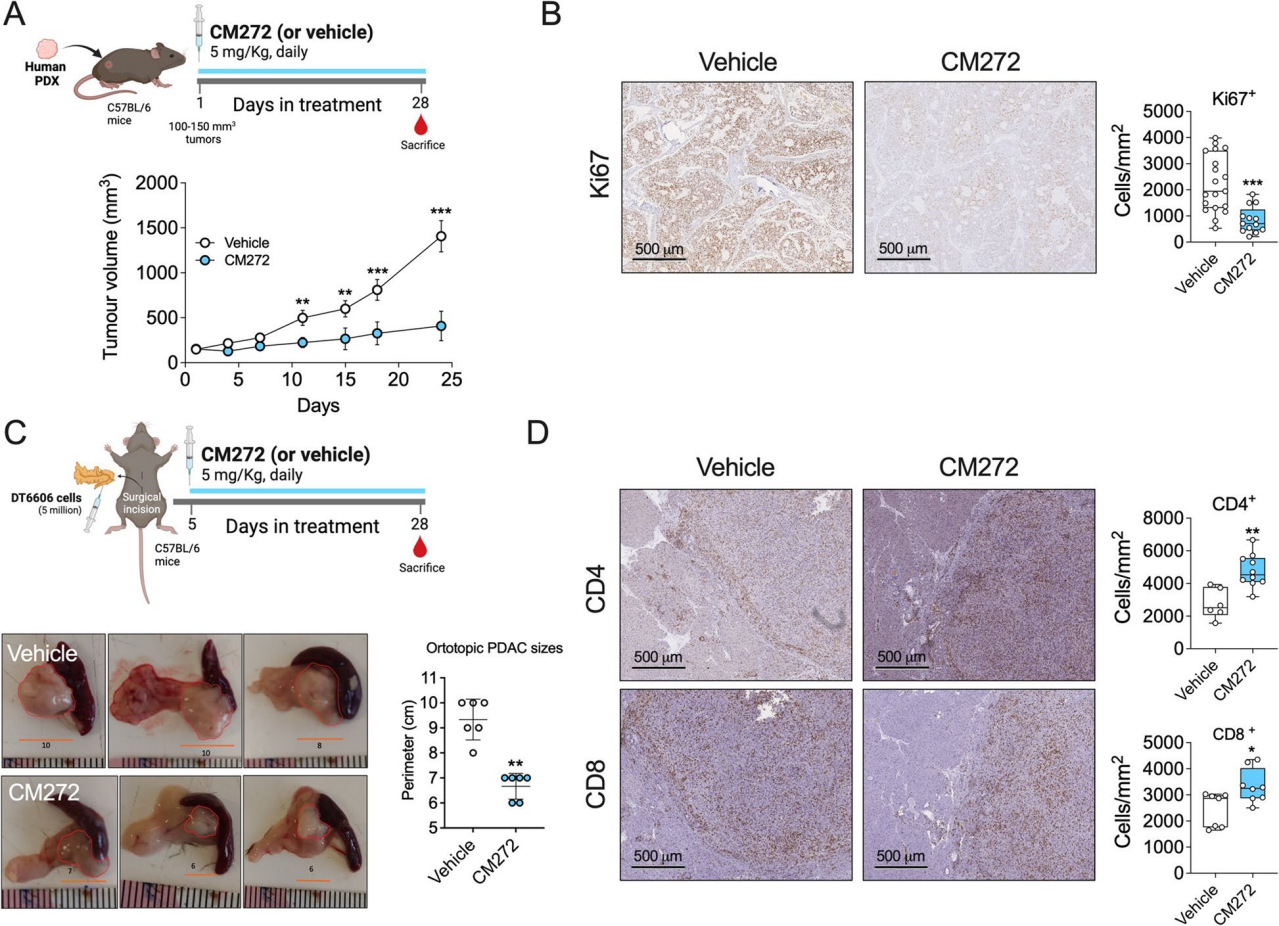
In vivo antitumoral effects of CM272. **A** Diagram of the experimental protocol. Effect of CM272 treatment on the growth of PDAC PDX mice. **B** Representative images of the immunohistochemical analysis of Ki67 protein in mouse PDX tumor samples and quantification. **C** Diagram of the experimental protocol. Representative tumor images of tumors in the PDAC orthotopic model and tumor size in Vehicle and CM272-treated groups. **D** Representative images of the immunohistochemical analysis of CD4 and CD8 proteins in the orthotopic tumor tissue samples and their quantification. *p < 0.05; **p < 0.01; ***p < *0.001;* ns*: not significant*

To further examine the antitumoral properties of CM272 we next employed a human PDAC patient-derived tumor xenograft (PDX) mouse model which clinical relevance has been recently reported [47]. When tumors reached 80 mm^3^, mice were randomized and treated with CM272 or the corresponding vehicle as described in Fig. 6A. We could observe how tumor growth was significantly reduced from early times after the onset of the treatment, and tumor weights were significantly reduced at the end of treatment (Fig. 6A). A dramatic decrease in cell proliferation was observed in CM272-treated mice, as detected by immunohistochemical staining for Ki67 (Fig. 6B).

Although PDX models retain the three-dimensional architecture of the original tumor, as well as genetic characteristics and metastatic potential [17] facilitating the implementation of pharmacological studies [16, 18, 19], the microenvironmental characteristics of PDAC (i.e. stroma, extracellular matrix or immune system) are lacking. Therefore, we also implemented a syngeneic allograft model in fully immunocompetent C57BL/6 mice by orthotopic injection of mouse PDAC cells directly into the pancreas. The selected cell line, DT6606 cells, has been derived from LSL-KrasG12D/ + ; Pdx-1-Cre (KC) mice in a C57BL/6 background [48]. As shown in Fig. 6C, CM272 administration started 7 days after DT6606 cells implantation, and after four weeks of treatment the size of tumors in animals receiving the drug was significantly lower than in controls. In agreement with our previous studies [23, 25, 26, 28], we did not observe any signs of toxicity in CM272-treated animals, and mice weights were normal at the end of treatment. No changes were observed in transaminases (ALT and ASL), lipase, or amylase serum levels between the groups, indicating the absence of hepatic and pancreatic injury (Fig. S7A). Consistently with our in vitro findings on the ability of CM272 to enhance PDAC cells immunogenicity (Fig. 4A), we observed a marked infiltration of CD4 + and CD8 T + cells in the tumour tissues of CM272-treated animals (Fig. 6D).

**Fig. 7:**
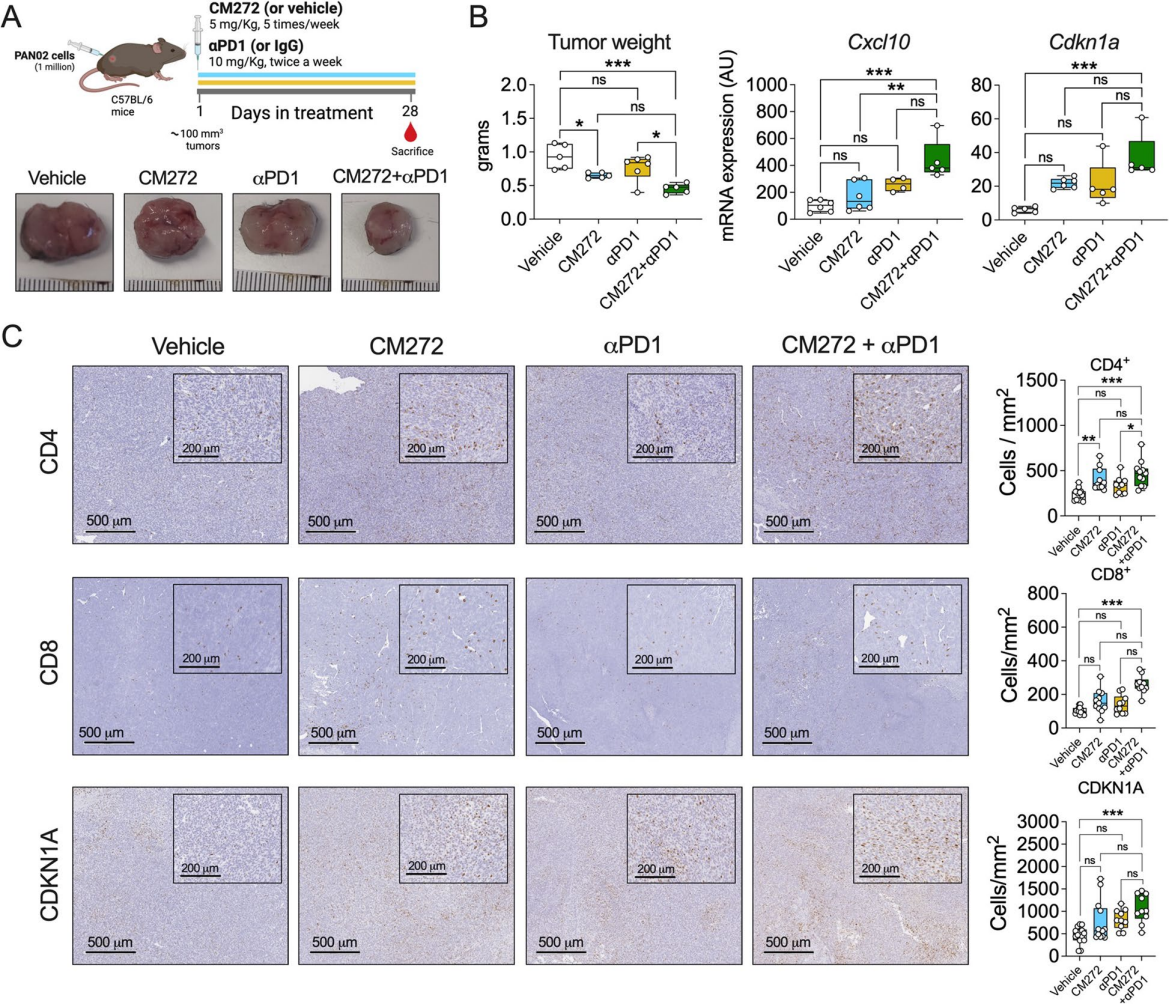
In vivo antitumoral effects of CM272 and its combination with immunotherapies. **A** Diagram of the experimental protocol. Representative tumor images and tumor weights of the subcutaneous PDAC model in which mice were treated with vehicle, CM272, anti-PD-1 and the combination of CM272 and anti-PD-1. **B** qPCR analysis of the expression of *Cxcl10* and *Cdk1a* genes in in tumor tissues from mice treated with vehicle, CM272, α-PD1 and the combination of CM272 and α-PD1. **C** Representative images showing the immunohistochemical detection of CD4, CD8 and CDKN1A and their quantification in tumors from mice treated with vehicle, CM272, α-PD1 and the combination of CM272 and α-PD-1. *p < 0.05; **p < 0.01; ***p < *0.001;* ns*: not significant*

In view of these findings, we decided to implement a second orthotopic syngeneic allograft model in immunocompetent mice, this time using the murine PDAC PAN02 cell line, known to be highly immunosuppressive and resistant to ICI-based therapy [49, 50]. In this model, CM272 treatment resulted in a moderate antitumoral effect, while anti-PD1 monoclonal antibody administration caused a minor decrease in tumour weight without statistical significance (Fig. 7A). As in the previous experimental model, transaminases, lipase, and amylase serum levels did not change between the groups (Fig. S7B). We confirmed the on-target effects of CM272 by the observation of a significant decrease in H3K9me2 staining in tumor tissues from mice treated with the inhibitor (Fig. S7C). Remarkably, the combination treatment including CM272 and anti-PD1 antibodies led to a significant tumour regression (Fig. 7B). Gene expression analyses in tumor tissues showed significantly increased mRNA levels of *Cxcl10* and *Cdkn1a* in the combination treatment group (Fig. 7C), in line with the in vitro responses to CM272 in cultured PDAC cells. Moreover, we also observed a significant elevation in the numbers of tumor-infiltrating CD4 + and CD8 + T cells in the combination group (Fig. 7D). Together, these results suggest that targeting the G9a/DNMT1/UHRF1 complex may be a new strategy to increase the response to immunotherapy in PDAC.

## Discussion

PDAC is an exceptionally aggressive cancer with a notably poor prognosis and a rising incidence. The disease’s aggressive nature, combined with the fact that the majority of patients are diagnosed at advanced or metastatic stages, makes the development of new therapeutic strategies for PDAC one of the most critical challenges in modern oncology. Despite numerous advancements in pharmacology and the introduction of new drugs, PDAC remains particularly resistant to current oncological treatments. While targeted therapies have become standard treatments for other cancers such as breast, lung, and colorectal cancer, PDAC continues to primarily depend on chemotherapy as its main treatment option. Due to the aggressive nature of PDAC, which often results in a rapid decline in the patient's health, there is an urgent need for more effective and better-tolerated therapeutic options. The major barriers to effective treatment of PDAC include its intra-tumoral heterogeneity, dense desmoplastic stroma, and immunosuppressive tumor microenvironment [51].

Recent research has highlighted the significant role that dysregulated epigenetic mechanisms play in the biology and heterogeneity of PDAC, contributing to disease progression, metastasis, and resistance to chemotherapy [10, 12, 18]. As a result, clinical trials have been initiated to assess the safety and efficacy of epigenetic therapies in PDAC patients [20]. However, the results observed so far suggest that, similar to chemotherapy, targeting epigenetic alterations as a monotherapy has not yet shown significant benefits in PDAC. If epigenetic drugs could show efficacy in selected patient subgroups and if their underlying mechanisms are able to confer tumor cell selectivity to these inhibitors, remain largely unclear.

In this study, we aimed to explore the roles of DNMT1, G9a, and UHRF1 proteins in this tumor by evaluating their expression in human PDAC tissue specimens. To this end, we employed a semi-quantitative scoring system similar to the one described by Abu-Alainin et al. for UHRF1 assessment in pancreatic cancer [31]. This approach allowed us to discern varying levels of staining corresponding to different degrees of protein expression. Notably, positivity for these three proteins was also observed in stromal tissue and normal pancreatic ducts in most cases, indicating a baseline expression level. To stratify tissue samples according to protein expression in the different cellular compartments, we categorized nuclear staining for DNMT1, G9a, and UHRF1 into negative-moderate weak (scores 0, 1 + , 2 +) or strong positivity (scores 3 +). Proteins were classified as over-expressed if their immunohistochemical staining was scored as 3 + . Using this method, we demonstrated the over-expression of DNMT1, G9a, and UHRF1 in PDAC cells compared to normal ducts. Importantly, a significant proportion of PDAC cases exhibited moderate staining for DNMT1, G9a, and UHRF1 within the stroma as well. The individual correlation of DNMT1, G9a and UHRF1 tumor expression with clinical-pathological variables revealed significant association with specific events, such as tumor stage or microvascular invasion. Furthermore, we demonstrated a strong and significant association between the concomitant over-expression of DNMT1, G9a and UHRF1 and the survival of the resected patients in two different cohorts of PDAC patients. The individual expression and potential role of DNMT1, UHRF1 and G9a in PDAC has been partially explored in previous studies [29–31, 33, 52, 53]. However, limited evidence is available on their prognostic significance. Given the functional and physical interaction of DNMT1 and G9a in DNA and histone methylation in numerous cancer-related cellular processes, as well as their binding with the regulator UHRF1, assessing their role as part of a functional complex, instead of as separated effectors, is clearly relevant. Our findings not only suggest that the simultaneous up-regulation of the three proteins retains a negative prognostic impact and is correlated with a more aggressive disease, but also that the concomitant pharmacological targeting of the epigenetic modifiers within this functional complex could represent a more effective therapeutic approach.

The rationale for the simultaneous inhibition of G9a and DNMT1 in PDAC was initially established by the synergistic antiproliferative action of combined G9a- and DNMT1-specific inhibitors, UNC0642 and AZA, respectively, in PDAC cells. We then tested a new class of small molecules designed to encompass both inhibitory activities, the dual inhibitor CM272. CM272 exerted a very potent antiproliferative effect in a wide panel of both human and mouse PDAC cell lines, as well as a reduction in their oncogenic capabilities. Interestingly, the compound was devoid of toxicity in non-tumoral pancreatic cells, both in vitro and in vivo. To elucidate the mechanisms underlying its activity, we performed transcriptomic studies in PDAC cells treated with the epigenetic compound. Changes in expression of apoptotic, oxidative stress and antiproliferative genes were the most outstanding. Potent induction of genes such as *ATF3, CDKN1A* or *BCL2L1*, was observed in PDAC cells treated with CM272, consistently leading to higher apoptosis levels. These observations align with previous studies showing that the independent pharmacological inhibition of G9a or DNMT1 in PDAC cell lines triggers senescence and autophagy [30, 33, 54–58]. Interestingly, it has been reported that *G9a* inactivation results in a transcriptional profile that is functionally antagonistic to that driven by oncogenic *KRAS*. Indeed, loss of *G9a* expression in the context of mutant *KRAS* led to increased levels of cell cycle regulatory molecules known to function as checkpoints to arrest cell growth in response to replication stress and to impair the EGFR-KRAS pathway [33]. The potent downregulation of EGFR protein levels in PDAC cells upon CM272 treatment confirms this observation. This effect may have important implications, considering the critical role that EGFR and its downstream pathways play in the initiation of pancreatic cancer [36, 37].

Epigenetic mechanisms, including DNA methylation and histone modifications, have also been involved in chemoresistance of pancreatic cancer cells via modulation of cell cycle- and apoptosis-related genes [59]. Consistent with these notions, and with our observations in CM272-treated cells, we found that this epigenetic inhibitor significantly increased the sensitivity to gemcitabine, as well as to cisplatin and FOLFIRINOX. These findings suggest that the simultaneous inhibition of the G9a/DNMT1 epigenetic complex can potentiate the efficacy of chemotherapy and could be considered for the design of more effective combination strategies with reduced systemic toxicity. While the detailed mechanisms of CM272-mediated “episensitization” to the effects of chemotherapy need to be further explored, the diverse pharmacological nature of the compounds tested together with CM272 suggest that fundamental processes involved in cancer cell survival might be involved.

Our transcriptomic analysis also identified changes in expression of metabolism-related genes. PDAC cells exhibit an extensive metabolic reprogramming which is directly involved in their malignant phenotype and supports sustained proliferation [60]. Among these metabolic adaptations, PDAC cells exhibit significant differences in the uptake, metabolism and utilization of nutrients. Consistent with our recent findings in pediatric liver cancer [26], we also observed that CM272 markedly reduced the expression of amino acid metabolic enzymes (*ASNS, PYCR1, BCAT2*), as well as genes involved in the serine pathway (*PHGDH, PSPH, PSAT1*). These routes supply both nitrogen and carbon sources fueling diverse biosynthetic pathways and contributing to energy production in pancreatic cancers [61, 62]. Activation of these metabolic routes has been associated with increased risk of PDAC progression, underscoring the emerging interest in developing new approaches to target these metabolic networks as cancer therapeutics [63, 64]. CM272-induced epigenetic reprogramming may thus lead to unique metabolic dependencies and vulnerabilities in tumor cells, suggesting that its combination with metabolism targeting drugs may offer new opportunities for a comprehensive treatment of pancreatic cancer. In this line, and according to the repression of sterol biosynthetic pathway elicited by CM272 in PDAC cells, the antiproliferative effect of lovastatin, an inhibitor of the rate-limiting enzyme in cholesterol synthesis, HMG-CoA reductase, was significantly increased by this epigenetic inhibitor. These observations support the interest of further exploring therapeutic combinations based on the ability of CM272 to counteract the pro-tumorigenic metabolic reprogramming of PDAC cells.

Interestingly, among the main transcriptomic responses to CM272 treatment in PDAC cells was a significant upregulation of genes involved in antigen processing and presentation pathways. Inhibition of the G9a/DNMT1 epigenetic complex resulted in the upregulation of HLA genes (*HLA-A, HLA-B, HLA-C*), as well as key genes involved in Class I MHC mediated antigen presentation such as *TAP1* and *B2M* [34]. Moreover, the expression of inflammatory chemokines potentially involved in the recruitment of cytotoxic T cells to the tumor microenvironment, like *CCL5* and *CCL10* [65], was also upregulated. Some of these effects have been previously described upon inhibition of epigenetic effectors like DNMT1 [54, 66]. In our study we provide genetic and pharmacological evidence indicating that the simultaneous inhibition of the G9a/DNMT1/UHRF epigenetic complex results in the strongest upregulation of these genes. These observations argue in favor of a beneficial combination of CM272 with ICI-based therapies. With this notion in mind, we went on to examine the safety and anti-PDAC activity of CM272 in the in vivo setting. First, we found a strong growth inhibitory effect of CM272 in an aggressive human PDAC PDX mouse model in the absence of signs of drug-related systemic or hepatic toxicity. However, as PDX models require immunodeficient mice to prevent immune rejection of the human derived xenografts, the CM272 antitumoral effect was validated in a syngeneic orthotopic PDAC mouse model. Importantly, treatment of these immunocompetent mice with CM272 markedly increased the infiltration of CD4 + and CD8 + T cells in the tumoral tissues. These, and our previous in vitro observations, led us to test a combination treatment including CM272 and α–PD1 antibodies in a second syngeneic mouse model more resistant to ICI-based therapies [50]. In this context, we clearly observed an enhanced antitumoral response in mice receiving the combination therapy compared to those receiving the single agents. This enhanced therapeutic response was accompanied by a robust immune infiltration of CD4 + and CD8 + T cell populations in the tumor tissues. These findings are in line with our previous observations in an immunocompetent mouse model of bladder carcinoma [67]. Additionally, in this PDAC model we also found a significant increase in the expression of CDK1A, suggesting that besides increasing tumor cells immunogenicity CM272 treatment could also induce cellular senescence [33]. This interesting double effect is being considered as a potential therapeutic strategy by recent studies, where delivery of innate immune agonists combined with senescence-inducing agents promotes T cell control of pancreatic cancer [68].

Taken together, our findings indicate that the simultaneous expression of G9a, DNMT1 and UHRF1 proteins in PDAC tissues represents a strong predictor of poor prognosis and survival. From a translational perspective, targeting of this epigenetic complex with specific inhibitors such as CM272 can induce a potent apoptotic response, reprogram key pro-tumorigenic metabolic routes in PDAC cancer cells, and enhances T-cell trafficking into the tumor microenvironment resulting in reduced tumor growth. As occurs with other epigenetic drugs, the antitumoral mechanisms of action of CM272 are likely multifarious. In this regard, given the strong pro-tumorigenic effects of the tumor stroma in PDAC [69, 70], we cannot discard that the antifibrogenic activity of CM272 previously described in liver tissue [28] could also play a mechanistic role here. Nevertheless, it will be interesting to investigate the effects of CM272 on the intratumoral immune microenvironment in other relevant genetic models of PDAC such as the KPC model [71], as well as its combination with other ICIs like anti-CTLA-4 antibodies.

## Conclusions

**Fig. 8:**
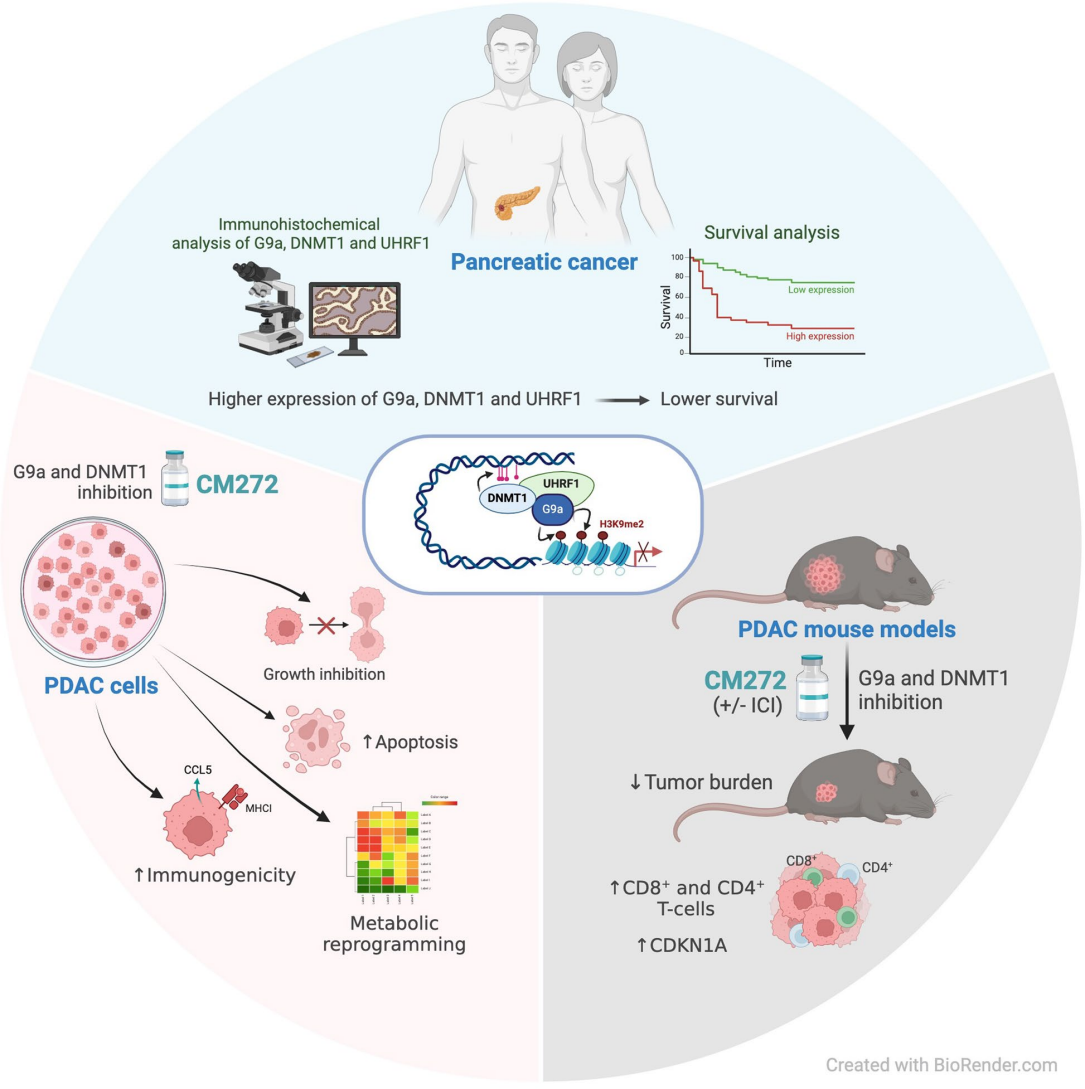
Overview of the main conclusions of the study. The combined overexpression of DNMT1, G9a, and UHRF1 in PDAC is a strong predictor of poor prognosis. CM272, by targeting this epigenetic complex, shows promising therapeutic potential by inducing apoptosis, reprogramming metabolic pathways, and enhancing immune responses. The combination of CM272 with immunotherapy offers a novel, effective treatment strategy for PDAC

Taken together, our observations underscore the potential of epigenetic compounds like CM272 for the treatment of this devastating disease. In combination with chemotherapeutic agents it may increase the limited efficacy of these conventional drugs. On the other hand, our promising results also suggest that pancreatic tumors could be sensitized to immune checkpoint inhibition by this epigenetic molecule. Given the lack of therapeutic options for patients with advanced disease, and the safety and efficacy demonstrated by CM272, we believe that additional preclinical studies in PDAC models should be pursued. In addition, the combined immunohistochemical analysis of G9a, DNMT1 and UHRF1 in PDAC tissue samples may be of prognostic value, and together with other clinical parameters could help to predict treatment response and disease progression (Fig. 8).

## Supplementary Information

Additional file 1: Supplementary Table 1. Supplementary Table 2. Supplementary Table 3. Supplementary Table 4.

Additional file 2: Supplementary Figure Legends.

Additional file 3: Figure S1. Figure S2. Figure S3. Figure S4. Figure S5. Figure S6. Figure S7.

## Data Availability

The data and material generated and analyzed during this study are included in the published article and its supplementary information files. Access to the data and material generated and analyzed during this study can be requested from the corresponding authors, who will consider all reasonable requests.

## Abbreviations

PDAC: Pancreatic ductal adenocarcinoma
OS: Overall survival
DFS: Disease-free survival
ICI: Immune checkpoint inhibitors
MSI-H: Microsatellite instability-high
TME: Tumor microenvironment
EHMT2: Euchromatic histone-lysine methyltransferase-2
DNMT1: DNA methyltransferase 1
UHRF1: Ubiquitin-like with PHD and RING finger domains 1
FFPE: Formalin-fixed, paraffin-embedded
DMEM: Dulbecco's Modified Eagle Medium
FBS: Fetal Bovine Serum
RPMI: Roswell Park Memorial Institute
ATCC: American Type Culture Collection
DMSO: Dimethyl sulfoxide
CI: Combination index
AZA: 5-Azacytidine)
5-FU: 5-Fluorouracil
dNTP: Deoxyribonucleic triphosphate
DTT: Dithiothreitol
PDX: Patient-derived xenograft
IHQ: Immunohistochemical
MHC: Major histocompatibility complex
GSEA: Gene-set enrichment analysis
EGFR: Epidermal growth factor receptor
Oxa: Oxaliplatin
