# Supplementary material for: Targeting of the G9a, DNMT1 and UHRF1 epigenetic complex as an effective strategy against pancreatic ductal adenocarcinoma
Source: J Exp Clin Cancer Res. 2025 Jan 15;44:13. doi: 10.1186/s13046-024-03268-5 (PMC11734372; doi:10.1186/s13046-024-03268-5)
Supplement: Supplementary file 1 — Additional file 1: Supplementary Table 1. Supplementary Table 2. Supplementary Table 3. Supplementary Table 4. [file 13046_2024_3268_MOESM1_ESM.pdf]

Supplementary Information

Additional file 1:

Supplementary Table 1. Characteristics of resected PDAC patients.

Characteristic	Italian Cohort Nº of patients (%) – n=42	Spanish Cohort Nº of patients (%) – n=49
Gender		
Male	26 (61.9)	30 (61.2)
Female	16 (38.1)	19 (38.8)
Sugery		
Duodenopancreatectomy	28 (66.7)	44 (89.8)
Distal pancreatectomy	14 (33.3)	5 (10.2)
Site		
Head	35 (83.3)	45 (91.8)
Body	5 (11.9)	1 (2.0)
Tail	2 (4.8)	3 (6.1)
Grade of differenciation		
1	2 (4.9)	4 (8.2)
2	23 (56.1)	28 (57.1)
3	16 (39.0)	17 (34.7)
Tumor (pT)		
1	4 (9.5)	0 (0.0)
2	29 (69.0)	1 (2.0)
3	9 (21.4)	48 (98.0)
Nodes (pN)		
0	17 (40.5)	15 (30.6)
1	12 (28.6)	34 (69.4)
2	13 (31.0)	0 (0.0)
Surgical margins		
R0	37 (90.2)	22 (44.9)
R1	4 (9.8)	27 (55.1)
Vascular Invasioin (pV)		
No	7 (23.3)	33 (68.8)
Yes	23 (76.7)	15 (31.2)
NA		0 (0.0)
Perineural Invasion (pPn)		
No	5 (14.3)	16 (33.3)
Yes	30 (85.7)	32 (66.7)
NA		
PDAC Relapse		
No	7 (16.7)	12 (24.5)
Yes	35 (83.3)	37 (75.5)
End of Surveillance		
Death	35 (83.3)	44 (89.8)
Alive	7 (16.7)	1 (2.0)
Alive (lost to follo-up)	0 (0.0)	4 (8.2)

Supplementary Table 2. IHQ tumor expression of G9a, DNMT1 and UHRF1 in Italian PDAC patient's cohort according to a semi-quantitative scoring system.

Scoring	G9a<br>Nº/n (%)<br>n=42	DNMT1<br>Nº/n (%)<br>n=42	UHRF1<br>Nº/n (%)<br>n=42
0	0 (0)	5 (11,9)	0 (0)
1+	2 (4,5)	13 (30,9)	6 (14,2)
2+	2 (4,5)	9 (21,4)	3 (7,1)
3+	37 (88)	9 (21,4)	32 (76,1)

Supplementary Table 3. G9a, DNMT1 and UHRF1 expression (3+) versus (2+/1+/0) in cancer tissue versus normal pancreatic ducts in the entire cohort (n=91) and in specific Italian and Spanish cohorts.

Whole Cohort	Normal duct n=91	Cancer n=91	OR (95% CI)	p-value
**DNMT1**				
0/+1/+2	70	44		
3	7	39	8.86 (3.65,21.55)	<0.001
**G9a**				
0/+1/+2	57	27		
3	12	63	11.08 (5.14, 23.90)	<0.001
**UHRF1**				
0/+1/+2	69	46		
3	1	44	66 (8.78, 496)	<0.001

Italian Cohort	Normal duct n=42	Cancer n=42	OR (95% CI)	p-value
**DNMT1**				
0/+1/+2	27	27		
3	4	9	2.25 (0.62, 8.20)	0.21
**G9a**				
0/+1/+2	18	4		
3	3	37	55.5 (11.21, 274.73)	<0.001
**UHRF1**				
0/+1/+2	20	9		
3	1	32	71.11 (8.36, 604.52)	<0.001

Spanish Cohort	Normal duct n=49	Cancer n=49	OR (95% CI)	p-value
**DNMT1**				
0/+1/+2	43	17		
3	3	30	125.36 (24.59, 639.12)	<0.001
**G9a**				
0/+1/+2	39	23		
3	9	26	4.904 (1.96, 12.25)	<0.001
**UHRF1**				
0/+1/+2	49	37		
3	0	12		

Supplementary Table 4. Correlation of DNMT1, G9a and UHRF1 expression in cancer and normal pancreatic ducts with clinicopathological variables.

	DNMT1			G9a			UHRF1		
**Variables**	**0/+1/+2 (n=44)**	**3 (n=39)**	**p-value**	**0/+1/+2 (n=27)**	**3 (n=63)**	**P Value**	**0/+1/+2 (n=46)**	**3 (n=44)**	**p-value**
**Gender**			0.8243			0.8135			0.7006
Male	27 (54.0)	23 (46.0)		17 (30.9)	38 (69.1)		29 (52.7)	26 (47.3)	
Female	17 (51.5)	16 (48.5)		10 (28.6)	25 (71.4)		17 (48.6)	18 (51.4)	
**Site**			0.6735			0.8610			0.6261
Head	38 (52.8)	34 (47.2)		23 (29.1)	56 (70.9)		41 (51.9)	38 (48.1)	
Body	4 (66.7)	2 (33.3)		2 (33.3)	4 (66.7)		2 (33.3)	4 (66.7)	
Tail	2 (40.0)	3 (60.0)		2 (40.0)	3 (60.0)		3 (60.0)	2 (40.0)	
**Surgery**			0.4366			0.6932			0.7133
Duodenopancreatectomy	33 (50.8)	32 (49.2)		22 (31.0)	49 (69.0)		37 (52.1)	34 (47.9)	
Distal pancreatectomy	11 (61.1)	7 (38.9)		5 (26.3)	14 (73.7)		9 (47.4)	10 (52.6)	
**Grade of differentiation**			0.9593			0.0499			0.2008
G1	17 (54.8)	14 (45.2)		13 (40.6)	19 (59.4)		20 (62.5)	12 (37.5)	
G2	19 (51.4)	18 (48.6)		7 (17.1)	34 (82.9)		17 (41.5)	24 (58.5)	
G3	8 (53.3)	7 (46.7)		7 (41.2)	10 (58.8)		9 (52.9)	8 (47.1)	
**Tumor (pT)**			**0.0092**			**0.0114**			**0.0002**
T1	2 (50.0)	2 (50.0)		0	4 (100)		1 (25.0)	3 (75.0)	
T2	19 (79.2)	5 (20.8)		4 (13.3)	26 (86.7)		7 (23.3)	23 (76.7)	
T3	23 (41.8)	32 (58.2)		23 (41.1)	33 (58.9)		38 (67.9)	18 (32.1)	
**Nodes (pN)**			0.0590			0.2232			**0.0380**
N0	18 (60.0)	12 (40.0)		8 (25.0)	24 (75.0)		15 (46.9)	17 (53.1)	
N1	18 (41.9)	25 (58.1)		17 (37.8)	28 (62.2)		28 (62.2)	17 (37.8)	
N2	8 (80.0)	2 (20.0)		2 (15.4)	11 (84.6)		3 (23.1)	10 (76.9)	
**pM**			0.9158			**0.0003**			0.2157
M0	16 (36.4)	28 (63.6)		20 (44.4)	25 (55.6)		35 (77.8)	10 (22.2)	
M1	1 (33.3)	2 (66.7)		3 (75.0)	1 (25.0)		2 (50.0)	2 (50.0)	
**Vascular Invasion (pV)**			**0.0440**			**0.0210**			**0.0067**
No	14 (37.8)	23 (62.2)		18 (45.0)	22 (55.0)		28 (70.0)	12 (30.0)	
Yes	21 (61.8)	13 (38.2)		7 (18.4)	31 (81.6)		15 (39.5)	23 (60.5)	
**Lymphatic Invasion**			0.8944			0.0020			0.7139
No	5 (38.5)	8 (61.5)		7 (50.0)	7 (50.0)		11 (78.6)	3 (21.4)	
Yes	12 (36.4)	21 (63.6)		15 (44.1)	19 (55.9)		25 (73.5)	9 (26.5)	
**Perineural Invasion (pPn)**			0.7393			0.3364			0.1148
No	10 (52.6)	9 (47.4)		9 (42.9)	12 (57.1)		14 (66.7)	7 (33.3)	
Yes	27 (48.2)	29 (51.8)		16 (25.8)	46 (74.2)		29 (46.8)	33 (53.2)	
**Adjuvant**			0.9574			0.9153			0.6194
No	10 (52.6)	9 (47.4)		7 (33.3)	14 (66.7)		10 (47.6)	11 (52.4)	
Yes	32 (53.3)	28 (46.7)		19 (29.2)	46 (70.8)		35 (53.8)	30 (46.2)	
