# Supplementary material for: Targeting of the G9a, DNMT1 and UHRF1 epigenetic complex as an effective strategy against pancreatic ductal adenocarcinoma
Source: J Exp Clin Cancer Res. 2025 Jan 15;44:13. doi: 10.1186/s13046-024-03268-5 (PMC11734372; doi:10.1186/s13046-024-03268-5)
Supplement: Supplementary file 2 — Additional file 2: Supplementary Figure Legends. [file 13046_2024_3268_MOESM2_ESM.docx]

**Supplementary Information**

**Additional file 2:**

**Figure legends**

**Figure S1.** (A) Growth inhibition curves of MIA PaCa-2 and PANC-1 cell lines treated with UNC0642 and AZA and the corresponding calculated GI_50_ values. (B) Growth inhibition curves of different human and mouse PDAC cell lines treated with CM272 and the corresponding calculated GI_50_ values.

**Figure S2.** (A) Most relevant GO functional categories of genes undergoing changes in expression identified by RNA-sequencing in PANC-1 cells treated with CM272 (GI_50_, 72 hours). (C) GSEA analysis of specific categories including heatmaps with a ranked list of genes modulated by CM272 from the RNA-seq data from PANC-1 cells treated with CM272.

**Figure S3.** (A) Viability of H6c7 cells treated with CM272 at two different concentrations (400nM and 800nM), gemcitabine (GEM), cisplatin (CDDP), FOLFIRINOX (FOLFX) and lovastatin (LVT) plus the combination of CM272 with all these compounds. (B) Evaluation of apoptosis in H6c7 cells treated with CM272 at two different concentrations (400nM and 800nM), gemcitabine (GEM), cisplatin (CDDP), FOLFIRINOX (FOLFX) and lovastatin (LVT) plus the combination of CM272 with all these compounds. Data are shown as Apoptosis *vs* Viability. ****p<0.001; ns: not significant vs* Control condition*.*

**Figure S4.** (A) qPCR analysis of *CCL5, HLA-A, HLA-B* and *HLA-C* expression in PANC-1 cells pretreated with CM-272 for 24 hours and then induced with IFNγ (100U/mL) for another 24 hours. (B) qPCR analysis of *B2M* and *TAP1* expression in MIA PaCa-2 (left) and PANC-1 (right) cells pretreated with CM-272 for 24 hours and then induced with IFNγ (100U/mL) for another 24 hours. **p<0.05; **p<0.01; ***p<0.001; ns: not significant.*

**Figure S5.** (A) qPCR analysis of *G9a, DNMT1* and *UHRF1* expression in MIA PaCa-2 (upper panel) and PANC-1cells (lower panel) transfected with G9a, DNMT1 or UHRF1-specific siRNAs, specific siRNAs combinations (siG9a + siDNMT1 and siG9a + SiDNMT1 + siUHRF1) and control siRNAs (siC) for 48 hours. (B) qPCR analysis of *CCl5, HLA-A, HLA-B* and *HLA-C* expression in PANC-1cells transfected with G9a, DNMT1 or UHRF1-specific siRNAs, specific siRNAs combinations (siG9a + siDNMT1 and siG9a + SiDNMT1 + siUHRF1) and control siRNAs (siC) for 48 hours. **p<0.05; **p<0.01; ***p<0.001; ns: not significant.*

**Figure S6.** (A) qPCR analysis of *CCL5, HLA-A, HLA-B* and *HLA-C* expression in PANC-1 cells treated with UNC, AZA, and the combination (UNC + AZA) at their GI_50_ during 72 hours. **p<0.05; **p<0.01; ***p<0.001;* ns*: not significant.*

**Figure S7.** (A) Serum levels of transaminases (ALT and AST), amylase (AMYL) and lipase (LIPC) from the orthotopic PDAC mouse model in mice treated with vehicle (Control) and CM272. (B) Serum levels of transaminases (ALT and AST), amylase (AMYL) and lipase (LIPC) from the subcutaneous PDAC model in mice treated with vehicle (Control), CM272, α-PD-1 and the combination of CM272 and α-PD-1. (C) Representative images showing the immunohistochemical detection of H3K9me2 mark in tumors from mice treated with vehicle, CM272, α-PD1 and the combination of CM272 and α-PD-1. ns*: not significant.*
