# Supplementary material for: Targeting of the G9a, DNMT1 and UHRF1 epigenetic complex as an effective strategy against pancreatic ductal adenocarcinoma
Source: J Exp Clin Cancer Res. 2025 Jan 15;44:13. doi: 10.1186/s13046-024-03268-5 (PMC11734372; doi:10.1186/s13046-024-03268-5)
Supplement: Supplementary file 3 — Additional file 3: Figure S1. Figure S2. Figure S3. Figure S4. Figure S5. Figure S6. Figure S7. [file 13046_2024_3268_MOESM3_ESM.pdf]

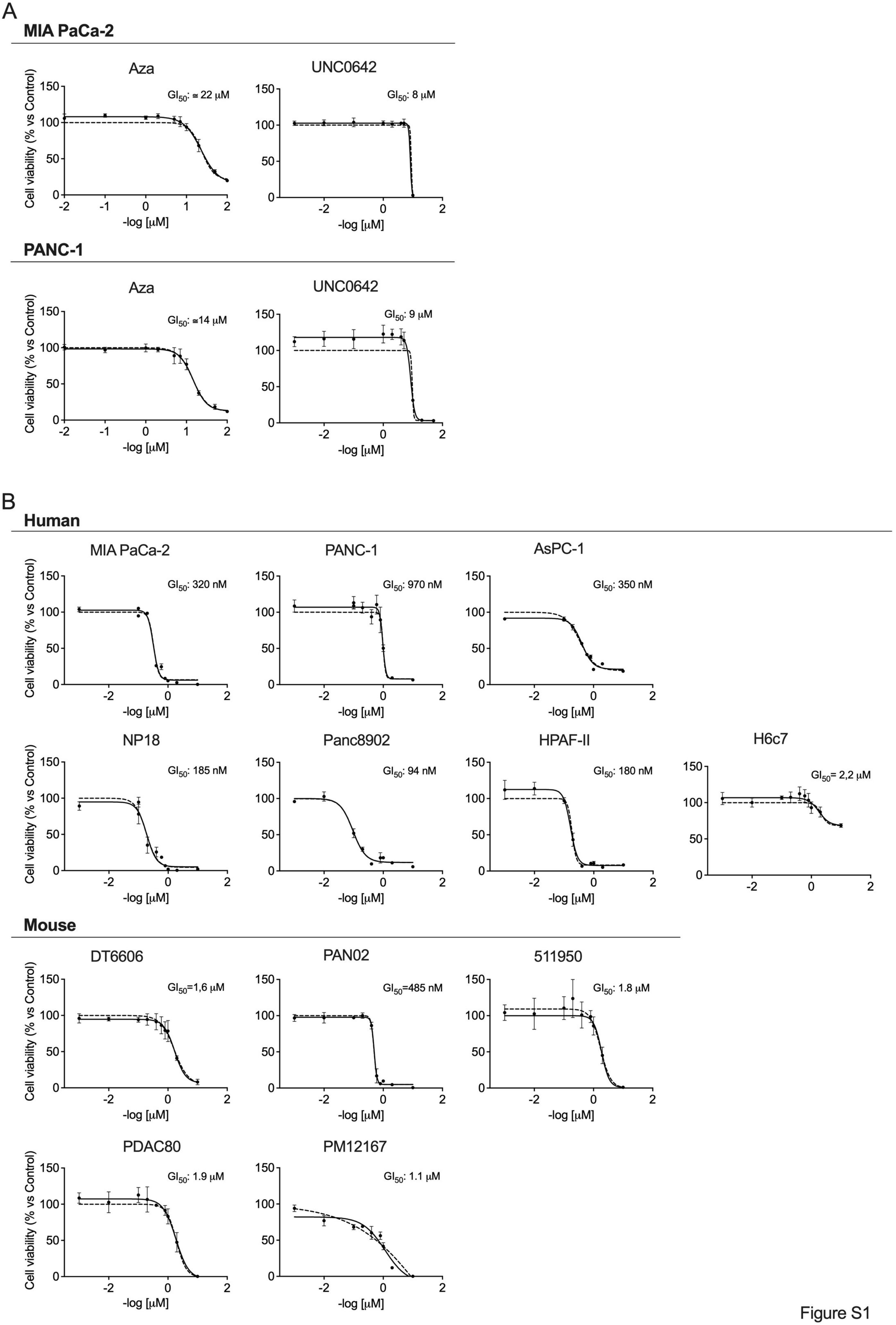
A
MIA PaCa-2
Aza
GI50: ≅ 22 µM
UNC0642
GI50: 8 µM
PANC-1
Aza
GI50: ≅14 µM
UNC0642
GI50: 9 µM
Cell viability (% vs Control)
-log [µM]
B
Human
MIA PaCa-2
GI50: 320 nM
PANC-1
GI50: 970 nM
AsPC-1
GI50: 350 nM
NP18
GI50: 185 nM
Panc8902
GI50: 94 nM
HPAF-II
GI50: 180 nM
H6c7
GI50= 2,2 µM
Mouse
DT6606
GI50=1,6 µM
PAN02
GI50=485 nM
511950
GI50: 1.8 µM
PDAC80
GI50: 1.9 µM
PM12167
GI50: 1.1 µM

Figure S1

A

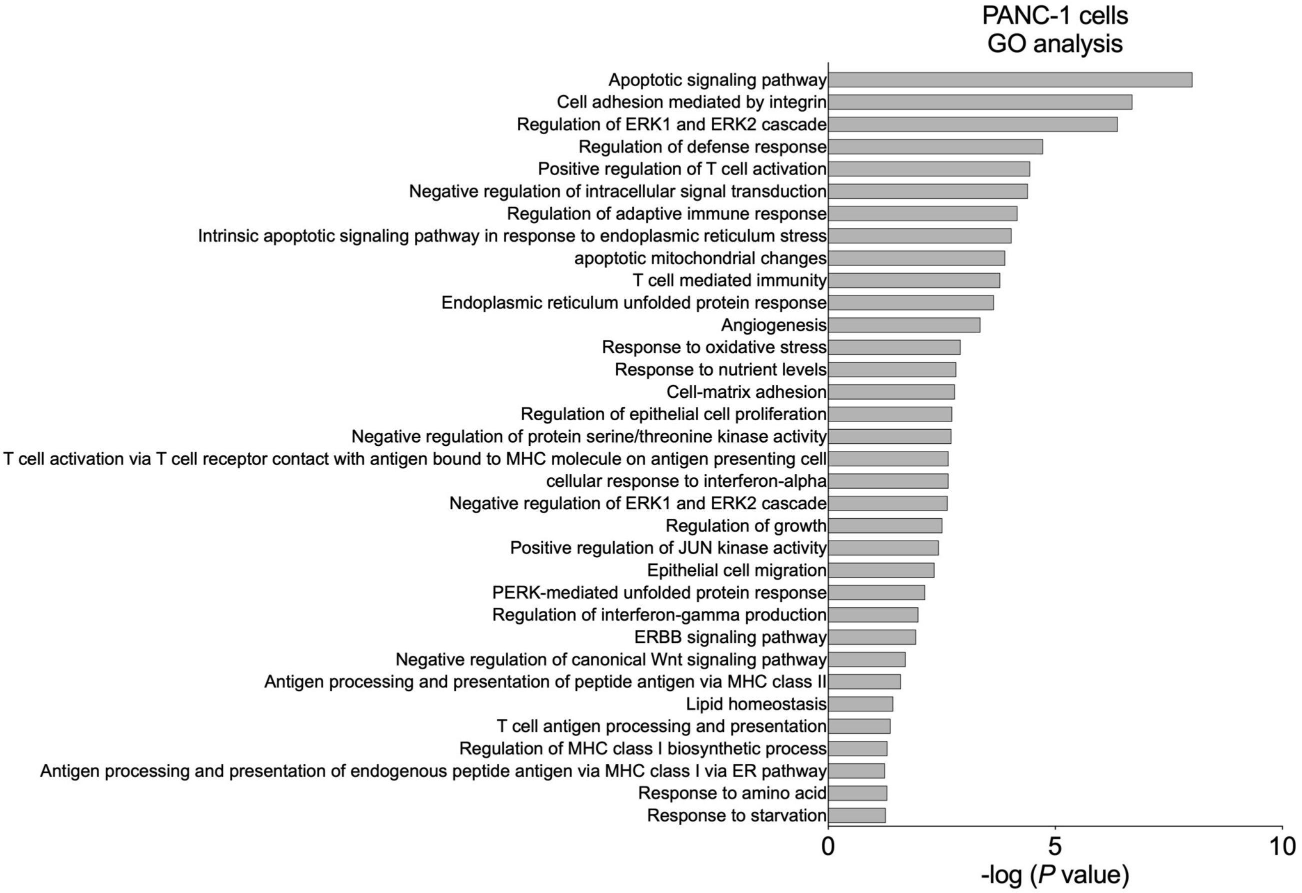
PANC-1 cells
GO analysis
Apoptotic signaling pathway
Cell adhesion mediated by integrin
Regulation of ERK1 and ERK2 cascade
Regulation of defense response
Positive regulation of T cell activation
Negative regulation of intracellular signal transduction
Regulation of adaptive immune response
Intrinsic apoptotic signaling pathway in response to endoplasmic reticulum stress
apoptotic mitochondrial changes
T cell mediated immunity
Endoplasmic reticulum unfolded protein response
Angiogenesis
Response to oxidative stress
Response to nutrient levels
Cell-matrix adhesion
Regulation of epithelial cell proliferation
Negative regulation of protein serine/threonine kinase activity
T cell activation via T cell receptor contact with antigen bound to MHC molecule on antigen presenting cell
cellular response to interferon-alpha
Negative regulation of ERK1 and ERK2 cascade
Regulation of growth
Positive regulation of JUN kinase activity
Epithelial cell migration
PERK-mediated unfolded protein response
Regulation of interferon-gamma production
ERBB signaling pathway
Negative regulation of canonical Wnt signaling pathway
Antigen processing and presentation of peptide antigen via MHC class II
Lipid homeostasis
T cell antigen processing and presentation
Regulation of MHC class I biosynthetic process
Antigen processing and presentation of endogenous peptide antigen via MHC class I via ER pathway
Response to amino acid
Response to starvation
0
5
10
-log (P value)

B

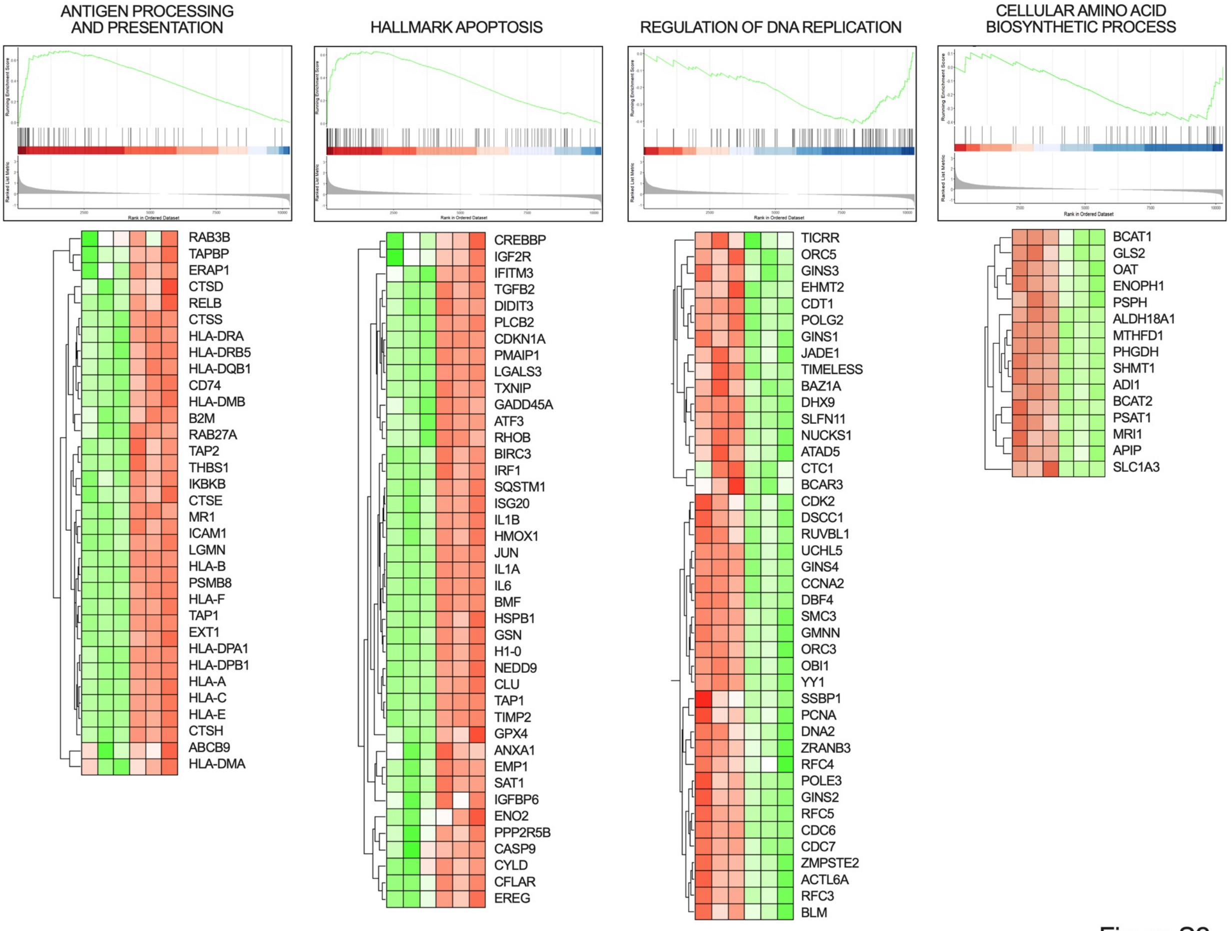
ANTIGEN PROCESSING AND PRESENTATION
HALLMARK APOPTOSIS
REGULATION OF DNA REPLICATION
CELLULAR AMINO ACID BIOSYNTHETIC PROCESS
RAB3B
TAPBP
ERAP1
CTSD
RELB
CTSS
HLA-DRA
HLA-DRB5
HLA-DQB1
CD74
HLA-DMB
B2M
RAB27A
TAP2
THBS1
IKBKB
CTSE
MR1
ICAM1
LGMN
HLA-B
PSMB8
HLA-F
TAP1
EXT1
HLA-DPA1
HLA-DPB1
HLA-A
HLA-C
HLA-E
CTSH
ABCB9
HLA-DMA
CREBBP
IGF2R
IFITM3
TGFB2
DIDIT3
PLCB2
CDKN1A
PMAIP1
LGALS3
TXNIP
GADD45A
ATF3
RHOB
BIRC3
IRF1
SQSTM1
ISG20
IL1B
HMOX1
JUN
IL1A
IL6
BMF
HSPB1
GSN
H1-0
NEDD9
CLU
TAP1
TIMP2
GPX4
ANXA1
EMP1
SAT1
IGFBP6
ENO2
PPP2R5B
CASP9
CYLD
CFLAR
EREG
TICRR
ORC5
GINS3
EHMT2
CDT1
POLG2
GINS1
JADE1
TIMELESS
BAZ1A
DHX9
SLFN11
NUCKS1
ATAD5
CTC1
BCAR3
CDK2
DSCC1
RUVBL1
UCHL5
GINS4
CCNA2
DBF4
SMC3
GMNN
ORC3
OBI1
YY1
SSBP1
PCNA
DNA2
ZRANB3
RFC4
POLE3
GINS2
RFC5
CDC6
CDC7
ZMPSTE2
ACTL6A
RFC3
BLM
BCAT1
GLS2
OAT
ENOPH1
PSPH
ALDH18A1
MTHFD1
PHGDH
SHMT1
ADI1
BCAT2
PSAT1
MRI1
APIP
SLC1A3

Figure S2

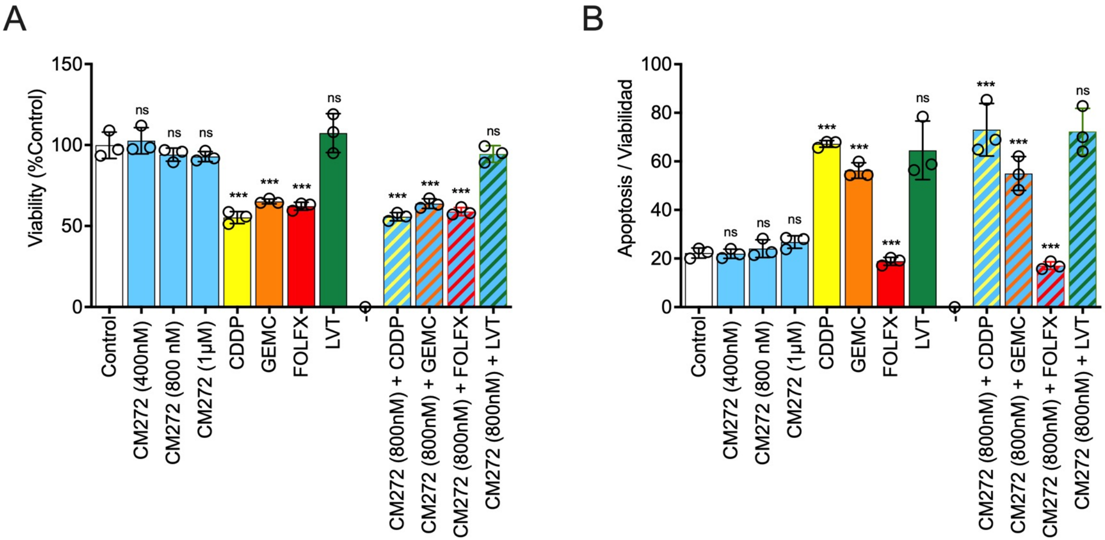
A
B
Viability (%Control)
Apoptosis / Viabilidad
ns
***
Control
CM272 (400nM)
CM272 (800 nM)
CM272 (1µM)
CDDP
GEMC
FOLFX
LVT
-
CM272 (800nM) + CDDP
CM272 (800nM) + GEMC
CM272 (800nM) + FOLFX
CM272 (800nM) + LVT

Figure S3

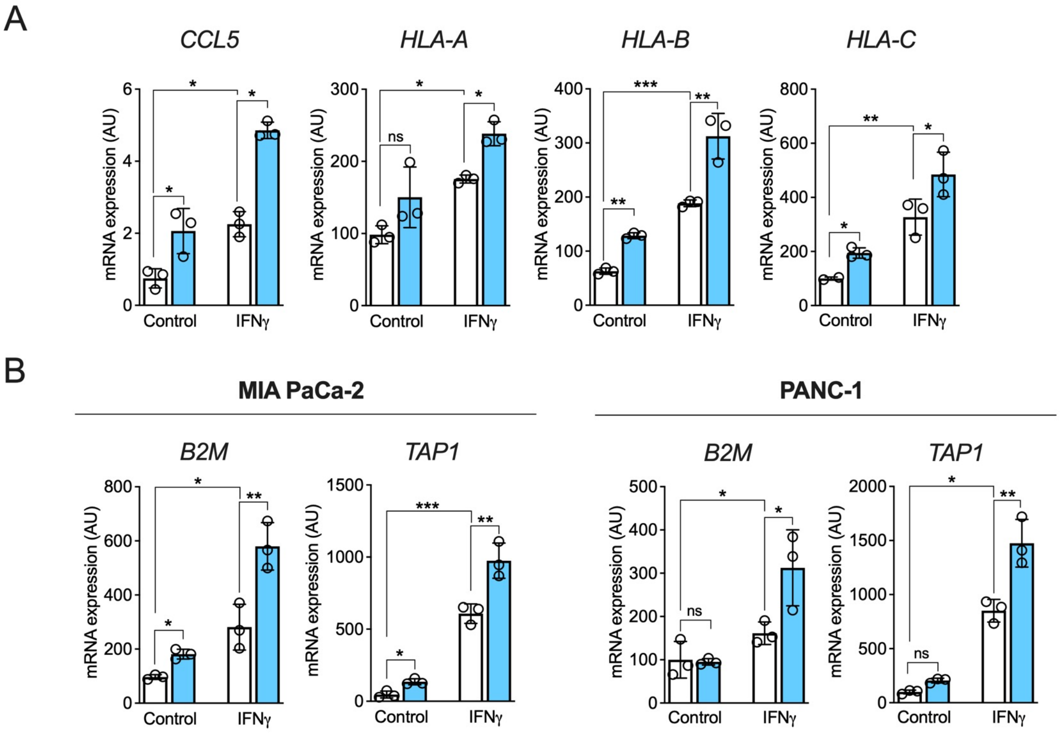
A
CCL5
HLA-A
HLA-B
HLA-C
mRNA expression (AU)
Control
IFNγ
ns
B
MIA PaCa-2
PANC-1
B2M
TAP1

Figure S4

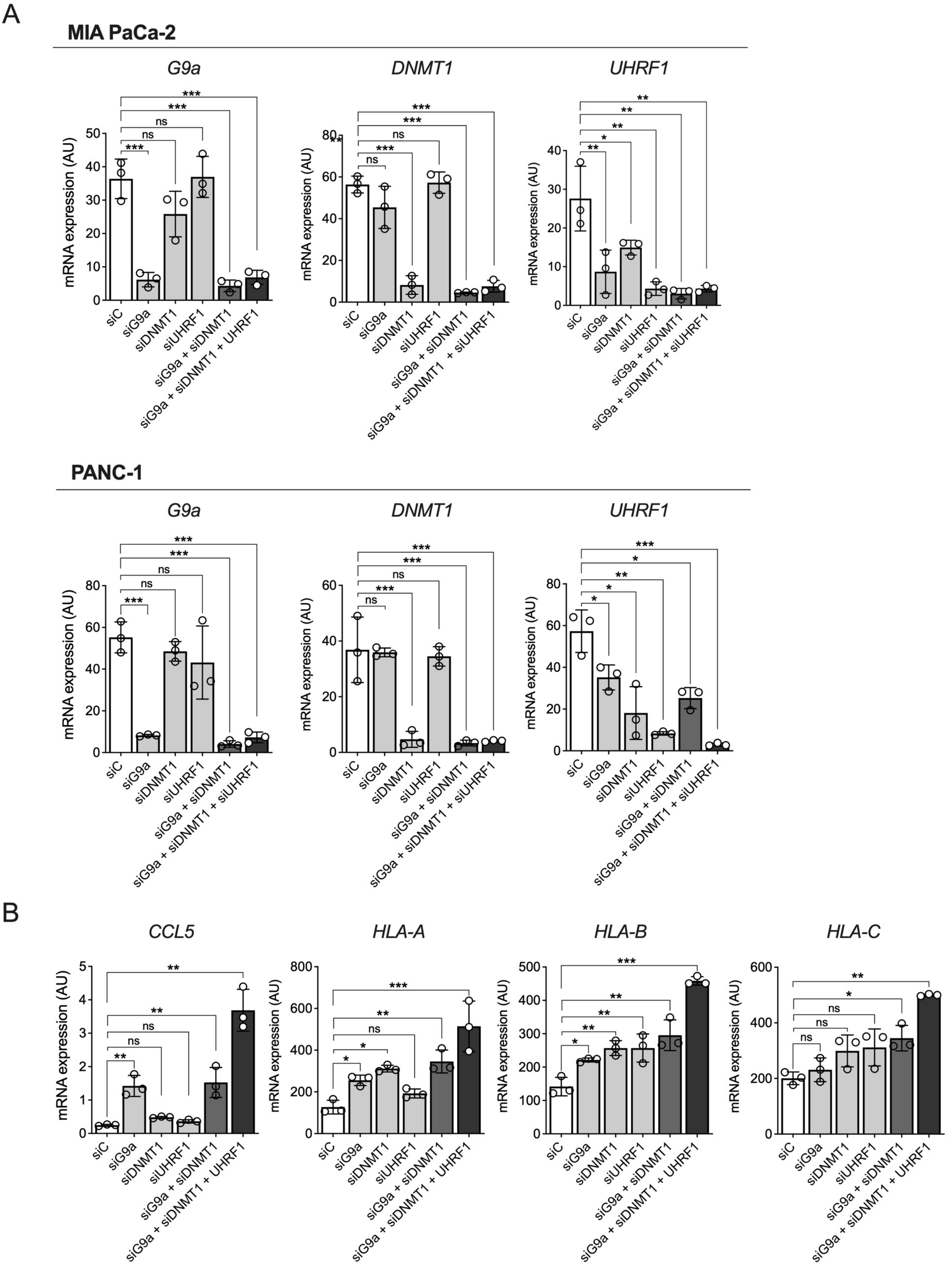
A
MIA PaCa-2
G9a
DNMT1
UHRF1
PANC-1
G9a
DNMT1
UHRF1
mRNA expression (AU)
siC
siG9a
siDNMT1
siUHRF1
siG9a + siDNMT1
siG9a + siDNMT1 + UHRF1
siG9a + siDNMT1 + siUHRF1
ns
*
**
***
B
CCL5
HLA-A
HLA-B
HLA-C

Figure S5

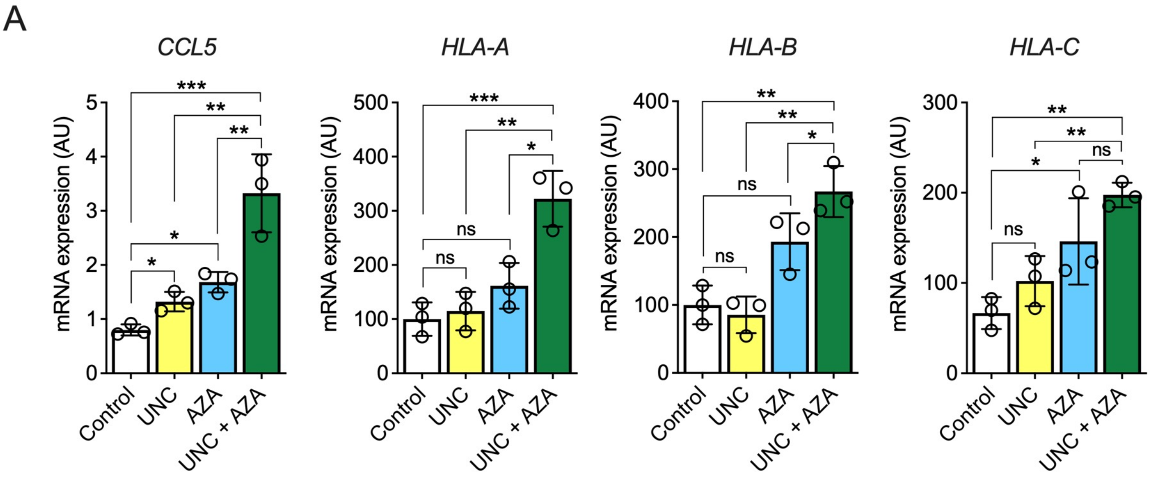
A
CCL5
HLA-A
HLA-B
HLA-C
mRNA expression (AU)
Control
UNC
AZA
UNC + AZA
ns
*
**
***

Figure S6

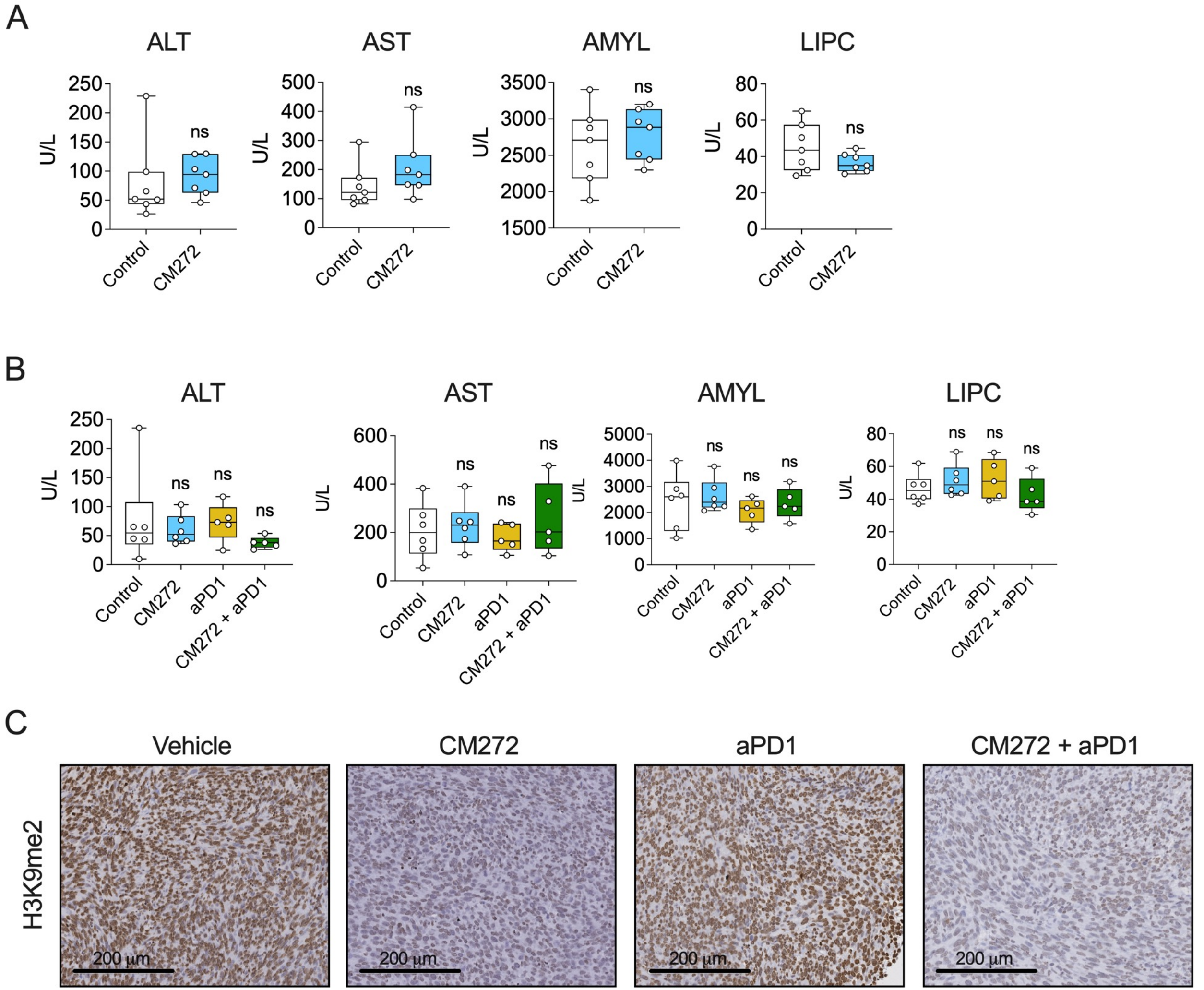
A
ALT
AST
AMYL
LIPC
U/L
ns
Control
CM272
B
ALT
AST
AMYL
LIPC
U/L
ns
Control
CM272
aPD1
CM272 + aPD1
C
Vehicle
CM272
aPD1
CM272 + aPD1
H3K9me2
200 µm

Figure S7
